# Supplementary material for: A penicillin-binding protein inhibitor series to target drug-resistant Neisseria gonorrhoeae
Source: Nat Microbiol. 2026 Apr 8;11(5):1348–60. doi: 10.1038/s41564-026-02309-3 (PMC13171604; doi:10.1038/s41564-026-02309-3)
Supplement: Supplementary file 1 — Supplementary Figs. 1–13, chemistry experimental details and Supplementary Tables 1–19. [file 41564_2026_2309_MOESM1_ESM.pdf]

nature microbiology

Supplementary information

https://doi.org/10.1038/s41564-026-02309-3

A penicillin-binding protein inhibitor series to target drug-resistant *Neisseria gonorrhoeae*

In the format provided by the authors and unedited

Supplementary Information

A penicillin-binding protein inhibitor series to target drug-resistant *Neisseria gonorrhoeae*

Tsuyoshi Uehara[1*], Allison L. Zulli[1‡], Brittany Miller[1‡], Lindsay M. Avery[1], Steven A. Boyd[1], Cassandra L. Chatwin[1], Guo-Hua Chu[1], Anthony S. Drager[1], Mitchell Edwards[1], Susan G. Emeigh Hart[1], Nathan J. Line[1], Cullen L. Myers[1], Gopinath Rongala[1], Annie Stevenson[1], Kyoko Uehara[1], Fan Yi[1], Bibo Wang[2], Zhenwu Liu[2], Mingyue Wang[2], Zhichao Zhao[2], Xinming Zhou[2], Haiyan Zhao[2], Caleb M. Stratton[3], Sandeepchowdary Bala[3], Christopher Davies[3], Rok Tkavc[4,5], Ann E. Jerse[5], Daniel C. Pevear[1,6], Christopher J. Burns[1], Denis M. Daigle[1], and Stephen M. Condon[1*]

[1]Venatorx Pharmaceuticals, Inc., Malvern, PA, USA

[2]BioDuro-Sundia, Beijing, China

[3]Department of Biochemistry & Molecular Biology, University of South Alabama, Mobile, AL, USA

[4]Henry M. Jackson Foundation for the Advancement of Military Medicine, Bethesda, MD, USA

[5]Department of Microbiology and Immunology, Uniformed Services University of the Health Sciences, Bethesda, MD, USA

[6]Spring Mill Pharma, Inc., Malvern, PA, USA

*Correspondence: tsuyoshi.uehara@gmail.com, stephencondon3418@gmail.com

‡These authors contributed equally.

Keywords: non-β-lactam, PBP inhibitor, boron, penicillin-binding protein, gonorrhea, antibiotic

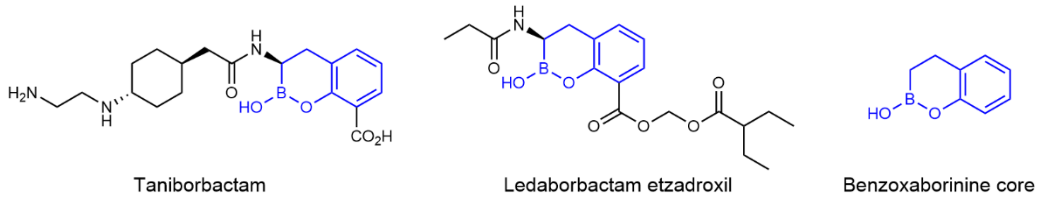

Figure S1. The chemical structures of the BLI taniborbactam and ledaborbactam etzadroxil. The benzoxaborinine core is shown in blue.

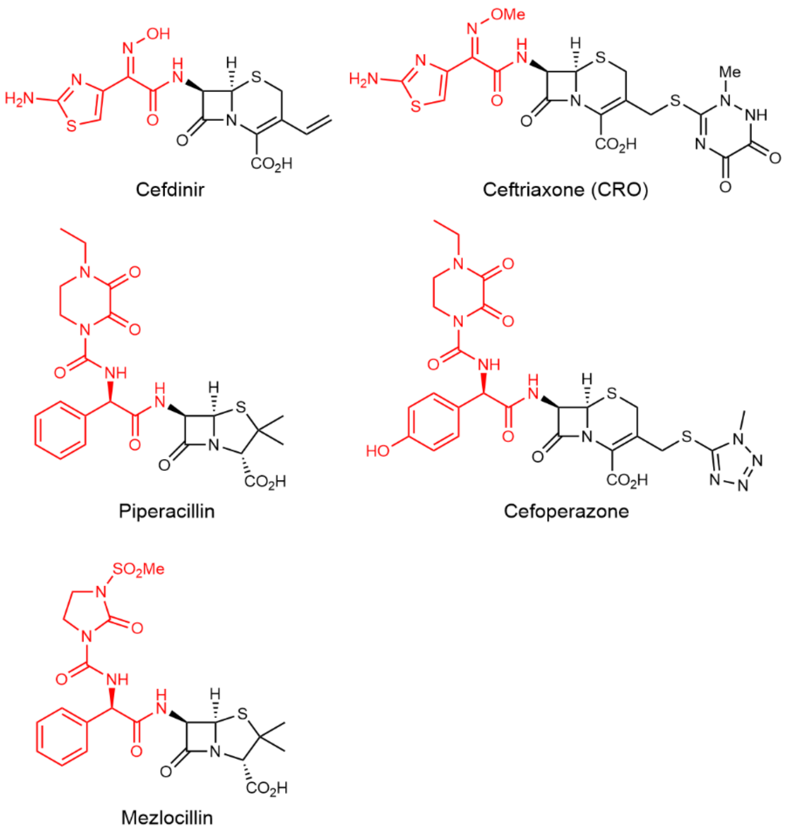

Figure S2. The chemical structures of cefdinir, ceftriaxone (CRO), piperacillin, cefoperazone, and mezlocillin. The β-lactam side chains are shown in red.

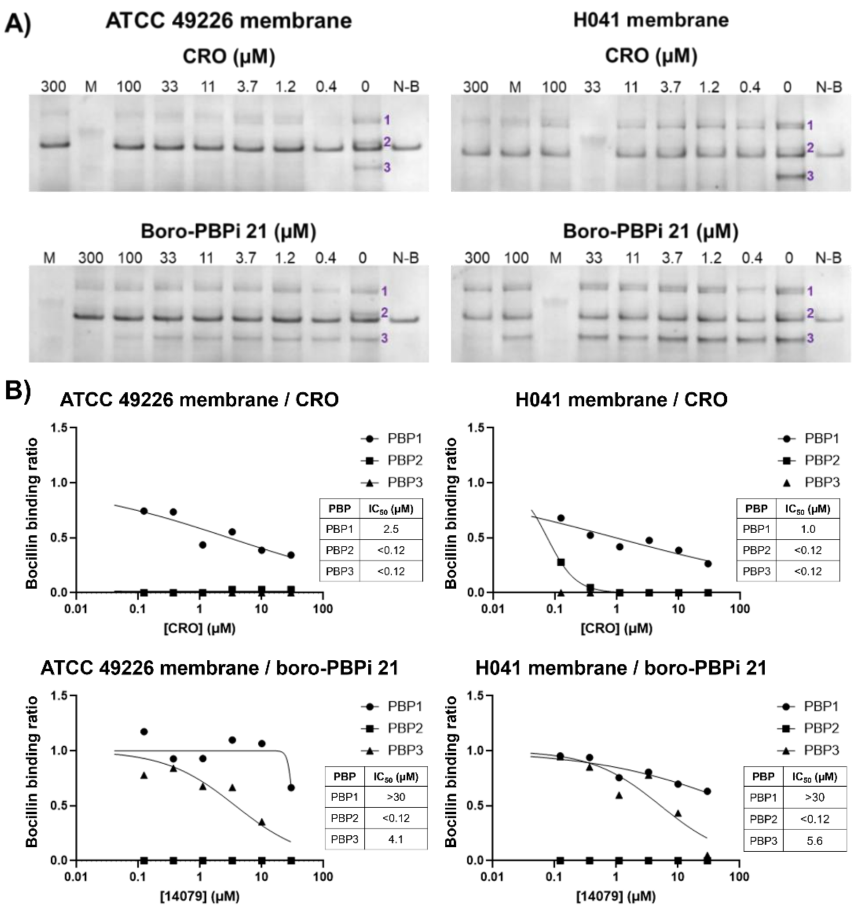

Figure S3. Binding of CRO and boro-PBPi **21** to PBP proteins in the membranes prepared from ATCC 49226 and H041.

The PBP binding assay was performed using membranes prepared from ATCC 49226 and H041 cells. The membranes were incubated with CRO or boro-PBPi **21** at the indicated concentrations at room temperature for 30 min, followed by incubation with 10 μM bocillin-FL for 30 min. **A)** Bocillin-FL-labeled PBPs were separated on an SDS-PAGE gel and visualized by fluorescence, as described in the Methods section. The bands of PBP1, PBP2, and PBP3 are labeled in purple. An autofluorescence band (no binding to β-lactams) in the samples without treatment with Bocillin-FL (indicated as "N-B") was observed near the band of PBP2, as described previously[1]. This non-specific fluorescent band had no apparent binding to **21** or CRO. The lanes labeled "M" stand for standard makers. The raw images of the gels are shown in **Figure S3b**. **B)** Plots of the intensity of the bands of Bocillin-FL-labeled PBPs to determine the IC_{50}s of each inhibitor. At 0.4 μM, PBP bands were excluded from quantification due to band lightness. At 0.12 μM, the binding of **21** to mosaic PBP2 was better than that of CRO in the H041 membrane, whereas both **21** and CRO bound tightly to non-mosaic PBP2 in the ATCC 49226 membrane. The binding affinity to PBP1 was lower than that for PBP2 for both inhibitors. At 0.12 μM, **21** did not bind PBP3, which is not essential for growth[2], whereas CRO bound tightly to it. Because PBP3 is not essential for the growth of *N. gonorrhoeae*[2], CRO binding to PBP3 is not expected to affect the MIC. The IC_{50}s of each inhibitor determined in the PBPs in the membrane-binding assays are shown.

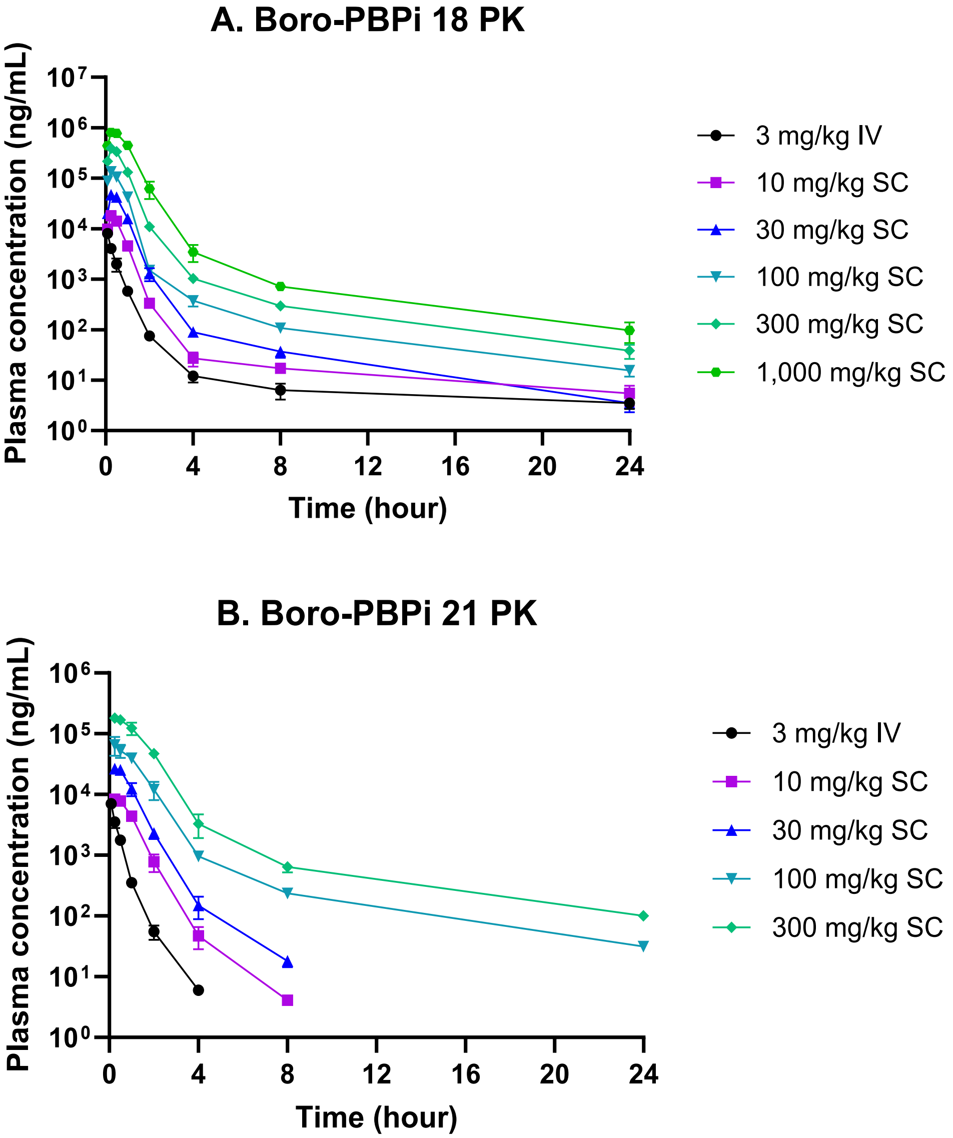

Figure S4. Plasma pharmacokinetics of boro-PBPi **18** (A) and **21** (B) following intravenous (IV) and subcutaneous (SC) administration in mice (n = 3 mice/group). The error bars show standard deviations. The pharmacokinetic parameters for **18** and **21** are shown in **Table S1** and **Table S2,** respectively.

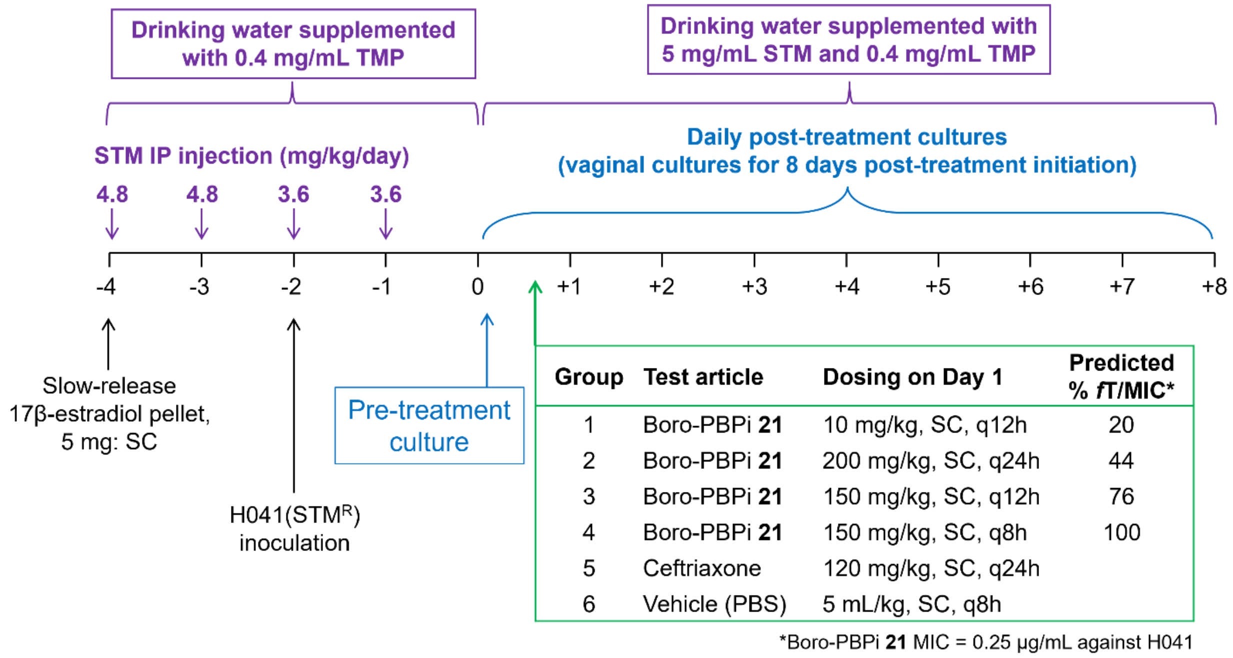

Group	Test article	Dosing on Day 1	Predicted % *f*T/MIC*
1	Boro-PBPi **21**	10 mg/kg, SC, q12h	20
2	Boro-PBPi **21**	200 mg/kg, SC, q24h	44
3	Boro-PBPi **21**	150 mg/kg, SC, q12h	76
4	Boro-PBPi **21**	150 mg/kg, SC, q8h	100
5	Ceftriaxone	120 mg/kg, SC, q24h	
6	Vehicle (PBS)	5 mL/kg, SC, q8h	

Figure S5. In vivo efficacy study design for boro-PBPi **21** in the murine vaginal infection model with ceftriaxone-resistant *N. gonorrhoeae* strain H041.

Abbreviations: IP, intraperitoneal; TMP, trimethoprim; SC, subcutaneous; STM, streptomycin.

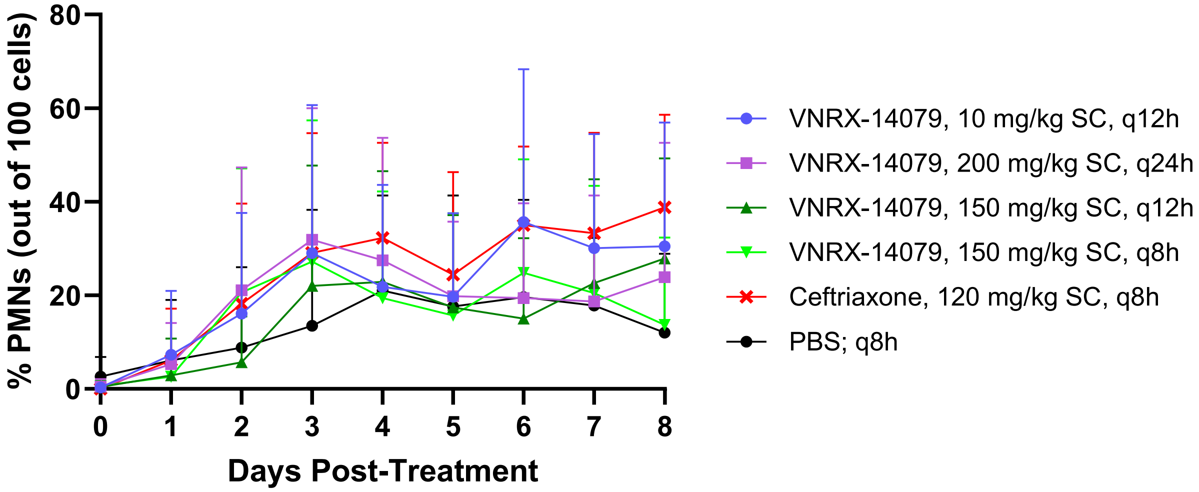

Figure S6. No difference in vaginal polymorphonuclear leukocytes (PMNs) over time in samples from the in vivo efficacy study for boro-PBPi **21**.

PMNs were counted in samples taken from the in vivo murein efficacy studies for **21** using the H041 strains. The data are plotted above. The comparison of groups using two-way ANOVA with repeated measures with Bonferroni post-hoc analysis did not show any significant differences (all *p*-values >0.05). The degrees of freedom and F values calculated from the comparison of groups were $F(5, 54) = 0.7214$.

The raw data are shown in **Table S5**.

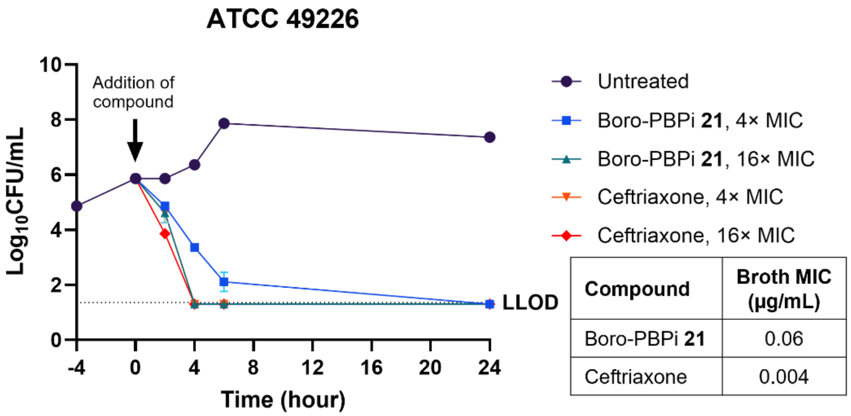

Compound	Broth MIC (μg/mL)
Boro-PBPi **21**	0.06
Ceftriaxone	0.004

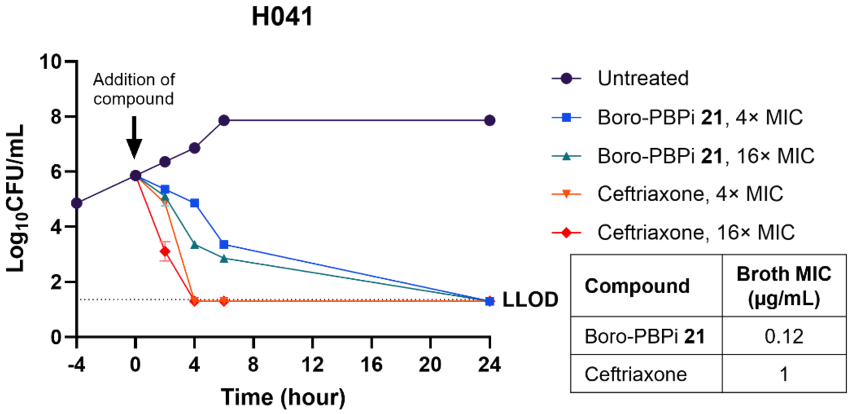

Compound	Broth MIC (μg/mL)
Boro-PBPi **21**	0.12
Ceftriaxone	1

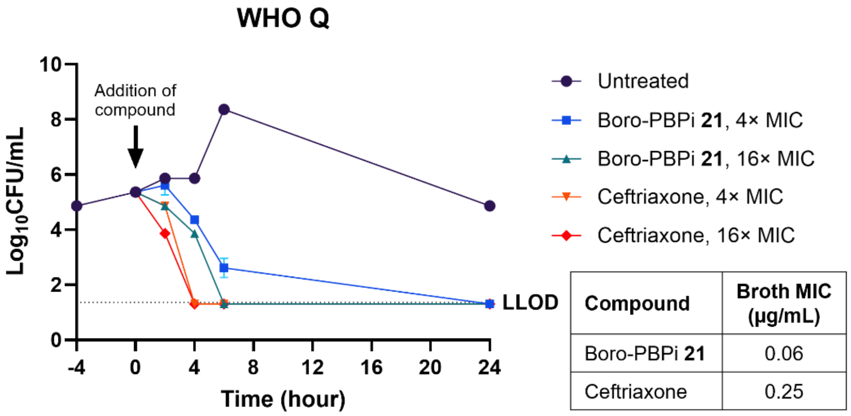

Compound	Broth MIC (μg/mL)
Boro-PBPi **21**	0.06
Ceftriaxone	0.25

Figure S7. In vitro bactericidal activity of boro-PBPi **21** and ceftriaxone in *N. gonorrhoeae* strains containing non-mosaic PBP2 (ATCC 49226) and mosaic PBP2 (WHO Q and H041).

The MICs were determined when the time-kill experiments were performed (*n*=2 biological replicates). LLOD: lower limit of detection. Data are presented as mean values ± SD.

CHEMISTRY EXPERIMENTAL DETAILS

Compound 1: The preparation of *Z*-(*R*)-3-(2-(2-aminothiazol-4-yl)-2-(hydroxyimino)-acetamido)-2-hydroxy-3,4-dihydro-2H-benzo[e][1,2]oxaborinine-8-carboxylic acid:

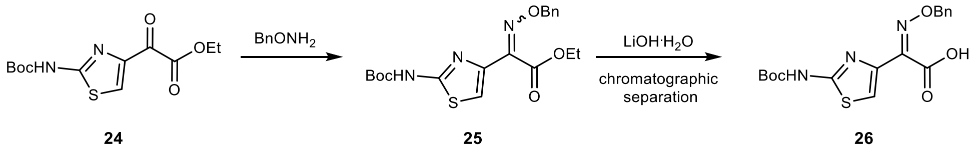

Scheme S1. The preparation of *O*-benzyl-protected oxyiminocarboxylic acid **26**.

Step 1: **Ethyl-2-((benzyloxy)imino)-2-(2-((*tert*-butoxycarbonyl)amino)thiazol-4-yl)acetate (23)**. To a solution of **24** (11 g, 36.7 mmol) in EtOH (500 mL), *O*-benzylhydroxylamine hydrochloride (10 g, 62.5 mmol) was added and the reaction mixture was stirred at ambient temperature. After 18 h, the reaction mixture was concentrated, and the residue was dissolved in dichloromethane (DCM), washed with saturated aqueous $NaHCO_3$, dried over anhydrous Na_2SO_4, filtered, and concentrated. The crude product was purified by flash silica gel chromatography (hexane/EtOAc, 20:1–2:1) to afford **25** as an inseparable mixture of *Z*- (major) and *E*- (minor) isomers (14.7 g, 36.3 mmol, quant.). Mass spectrum, ESI-MS *m/z* 406 $(M + H)^+$, calculated for $C_{19}H_{24}N_3O_5S$.

Step 2: **(*Z*)-2-((benzyloxy)imino)-2-(2-((*tert*-butoxycarbonyl)amino)thiazol-4-yl)acetic acid (26)**. To a solution of **25** (14.7 g, 36.3 mmol) in tetrahydrofuran (THF, 200 mL) and water (200 mL), LiOH hydrate (840 mg, 20 mmol) was added, and the reaction mixture was stirred at ambient temperature. After 2 h, LC/MS analysis showed complete hydrolysis of the less-hindered *E*-isomer. The reaction mixture was diluted with water and extracted with diethyl ether. The combined ether extracts were concentrated, and the residue was dissolved in THF (150 mL), MeOH (150 mL), and water (150 mL), treated with LiOH hydrate (4.41 g, 105 mmol), and stirred at ambient temperature. After 2 days, the reaction mixture was concentrated to remove the organic solvents and then acidified with 1 N HCl (pH 3). The solid was collected by filtration,

washed with water, and dried *in vacuo* to yield *Z*-**26** as a tan solid (9.3 g, 70% yield). Mass spectrum, ESI-MS *m/z* 378 $(M + H)^+$, calculated for $C_{17}H_{20}N_3O_5S$.

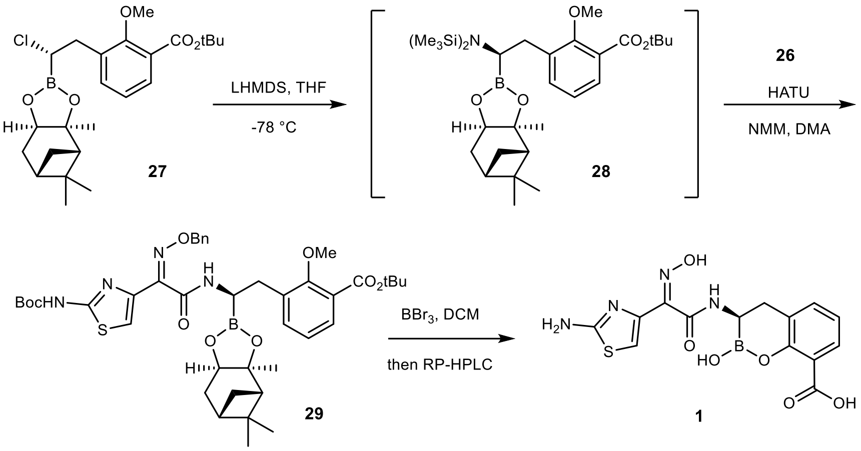

Scheme S2. The preparation of oxyimino-containing benzoxaborinine **1**.

Step 3: ***tert*-Butyl-3-((*R*)-2-((*Z*)-2-((benzyloxy)imino)-2-(2-((*tert*-butoxycarbonyl)amino)-thiazol-4-yl)acetamido)-2-((3a*S*,4*S*,6*S*,7a*R*)-3a,5,5-trimethylhexahydro-4,6-methanobenzo-[d][1,3,2]-dioxaborol-2-yl)ethyl)-2-methoxybenzoate (29)**. To a solution of **27** (1.35 g, 3.0 mmol) in THF (9 mL) at −78 °C, a solution of lithium hexamethyldisilazide (LHMDS, 3.0 mL, 1 M in THF, 3.0 mmol) was added dropwise. Following the addition, the cold bath was removed, and the reaction mixture was slowly warmed to ambient temperature. After 18 h, the resulting solution of **28** (*ca.* 3 mmol) was used without further purification.

To a mixture of **26** (3.3 mmol) and *N*-[(dimethylamino)-1*H*-1,2,3-triazolo-[4,5-*b*]pyridin-1-ylmethylene]-*N*-methylmethanaminium hexafluorophosphate *N*-oxide (HATU, 3.3 mmol) in *N,N*-dimethylacetamide (DMA, 9 mL) was added 4-methylmorpholine (NMM, 3.6 mmol). After 90 min, the previously prepared solution of **28** (3.0 mmol) was added. The resulting mixture was stirred for 2.5 h, diluted with EtOAc, washed successively with water and brine, dried over anhydrous Na_2SO_4, filtered, and concentrated. The crude residue was purified by flash silica gel chromatography (20−100% EtOAc/hexanes) to give **29** as an off-white-colored solid (50−70% yield). Mass spectrum, ESI-MS *m/z* 789 $(M + H)^+$, calc'd for $C_{41}H_{54}BN_4O_9S$.

Step 4: ***Z*-(*R*)-3-(2-(2-aminothiazol-4-yl)-2-(hydroxyimino)acetamido)-2-hydroxy-3,4-dihydro-2H-benzo[e][1,2]oxaborinine-8-carboxylic acid (1).** To a solution of **29** (0.4 mmol) in anhydrous DCM (15 mL) at -78 °C under argon, BBr_3 (1.0 M in DCM, 2.4-4 mmol) was added dropwise, and the reaction mixture was slowly warmed to 0 °C. After 1–2 h, the reaction mixture was quenched with water (2 mL) and methanol (20 mL), concentrated to remove DCM, and purified by preparative RP-HPLC using a Waters XBridge™ C18 column (5-40% acetonitrile (ACN) in water containing 0.1% TFA over 10 min; flow rate: 45 mL/min). The product-containing fractions were lyophilized to dryness to yield **1** as a white-colored solid (20–40% yield). ^1H NMR (MeOH-*d₄*): δ 7.89–7.78 (m, 1H), 7.40 (m, 1H), 7.04–6.83 (m, 1H), 6.13 (s, 1H), 3.45 (m, 1H), 3.04 (m, 2H). Mass spectrum, ESI-MS *m/z* 377 $(M + H)^+$, calc'd for $C_{14}H_{14}BN_4O_6S$.

Compound 2: The preparation of (3*R*)-3-(2-(4-ethyl-2,3-dioxopiperazine-1-carboxamido)-2-(4-hydroxyphenyl)-acetamido)-2-hydroxy-3,4-dihydro-2H-benzo[e][1,2]oxaborinine-8-carboxylic acid:

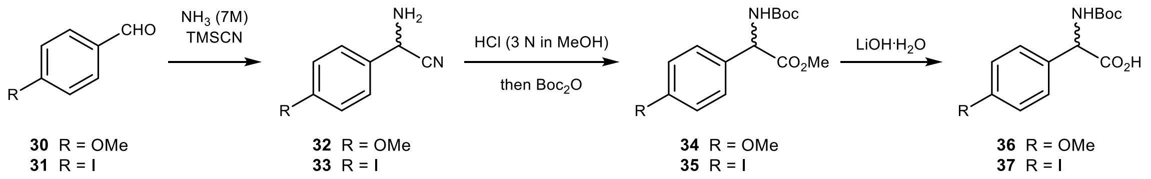

Scheme S3. The synthesis of racemic aryl glycine intermediates **36** and **37**.

Step 1: **2-((tert-butoxycarbonyl)amino)-2-(4-methoxyphenyl)acetic acid (36)** was prepared using the general procedures described for the preparation of *rac*-**53** (*vide infra*). Mass spectrum, ESI-MS *m/z* 282 $(M + H)^+$, calculated for $C_{14}H_{20}NO_5$.

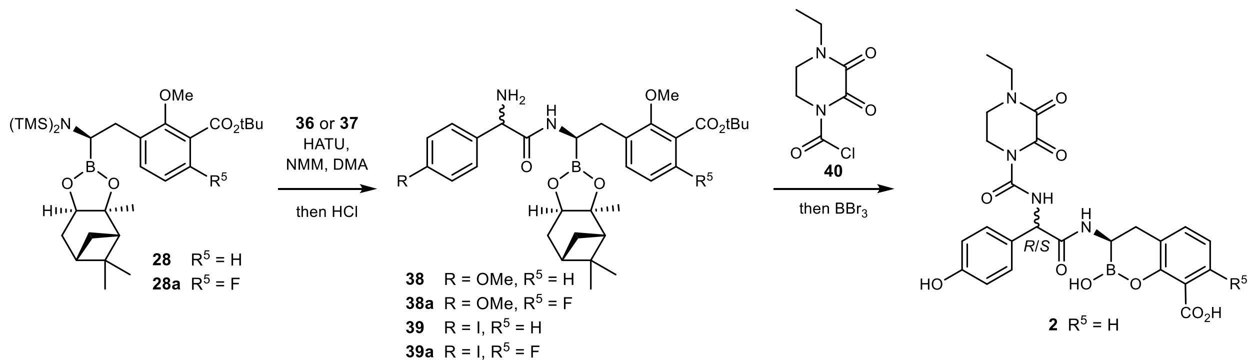

Scheme S4. Preparation of phenol-based boro-PBPi **2**.

Step 2: **2-(((*R*)-2-(3-(*tert*-butoxycarbonyl)-2-methoxyphenyl)-1-((3a*S*,4*S*,6*S*,7a*R*)-3a,5,5-trimethylhexa-hydro-4,6-methanobenzo[d][1,3,2]dioxaborol-2-yl)ethyl)amino)-1-(4-methoxyphenyl)-2-oxoethan-1-aminium (38)** was prepared using the general procedures described for the preparation of **43** (*vide infra*). Mass spectrum, ESI-MS *m/z* 593 $(M + H)^+$, calculated for $C_{33}H_{46}BN_2O_7$.

Step 3: **(3*R*)-3-(2-(4-ethyl-2,3-dioxopiperazine-1-carboxamido)-2-(4-hydroxyphenyl)-acetamido)-2-hydroxy-3,4-dihydro-2H-benzo[e][1,2]oxaborinine-8-carboxylic acid (2)**. To a solution of **36** (0.5 g, 0.9 mmol) in DCM (16 mL) was cooled to 0 °C. *N,N*-Diisopropylethylamine (DIPEA, 0.47 mL, 2.8 mmol) was added, followed by **38** (0.3 g, 1.4 mmol), and the reaction mixture was warmed to ambient temperature. After 0.5 h, the reaction mixture was poured into water and extracted with DCM. The layers were separated, and the organic phase was dried over anhydrous Na_2SO_4, filtered, and concentrated to give the ureido intermediate, which was used in the next step without further purification. Mass spectrum, ESI-MS *m/z* 761 $(M + H)^+$, calculated for $C_{40}H_{54}BN_4O_{10}$.

The crude ureido intermediate was treated with BBr_3 in DCM as described for the preparation of **1**, and the final product was purified by RP-HPLC [Waters™ XBridge C18 column (5-40% ACN in H_2O containing 0.1% TFA over 10 min; flow rate: 45 mL/min)]. The product-containing fractions were combined and lyophilized to dryness to afford **2** as a white-colored solid (20–40% yield). ^1H NMR (MeOH-*d*4): δ 9.42–9.20 (m, 1H), 7.82 (m, 1H), 7.38 (m, 1H), 7.05 (m, 1H), 6.80 (m, 2H),

6.50 (m, 2H), 5.30 (m, 1H), 3.98 (m, 2H), 3.60 (m, 2H), 3.50 (m, 2H), 3.32 (m, 1H), 2.98–2.80 (m, 2H), 1.20 (m, 3H) ppm. Mass spectrum, ESI-MS *m/z* 525 $(M + H)^+$, calculated for $C_{24}H_{26}BN_4O_9$.

Boro-PBPi **3–9** were prepared following the general methods outlined for the synthesis of **2**.

Compound 3: (*R*)-3-((*R*)-2-(4-ethyl-2,3-dioxopiperazine-1-carboxamido)-2-(3-fluoro-4-hydroxyphenyl)acetamido)-2-hydroxy-3,4-dihydro-2H-benzo[e][1,2]oxaborinine-8-carboxylic acid. ^{1}H NMR (MeOH-d_4): δ 9.25 (m, 1H), 7.80 (m, 1H), 7.38 (m, 1H), 7.00 (m, 1H), 6.75 (m, 2H), 6.55 (m, 1H), 5.38 (m, 1H), 3.98 (m, 2H), 3.62 (m, 2H), 3.50 (m, 2H), 3.30 (m, 1H), 2.99–2.75 (m, 2H), 1.20 (m, 3H). Mass spectrum, ESI-MS *m/z* 543 $(M + H)^+$, calculated for $C_{24}H_{25}BFN_4O_9$.

Compound 4: (*R*)-3-((*R*)-2-(4-ethyl-2,3-dioxopiperazine-1-carboxamido)-2-(3-fluoro-4-hydroxyphenyl)acetamido)-7-fluoro-2-hydroxy-3,4-dihydro-2H-benzo[e][1,2]oxaborinine-8-carboxylic acid. Prepared using intermediate **38a**. ^{1}H NMR (MeOH-d_4): δ 9.30 (m, 1H), 7.10 (m, 1H), 6.85 (m, 2H), 6.70 (m, 2H), 5.30 (m, 1H), 3.99 (m, 2H), 3.62 (m, 2H), 3.50 (m, 2H), 3.20 (s, 1H), 2.85–2.65 (m, 2H), 1.20 (m, 3H). Mass spectrum, ESI-MS *m/z* 561 $(M + H)^+$, calculated for $C_{24}H_{24}BF_2N_4O_9$.

Compound 5: (3*R*)-3-(2-(4-ethyl-2,3-dioxopiperazine-1-carboxamido)-2-(2-fluoro-4-hydroxyphenyl)acetamido)-2-hydroxy-3,4-dihydro-2H-benzo[e][1,2]oxaborinine-8-carboxylic acid. ^{1}H NMR (MeOH-d_4): δ 9.50–9.30 (m, 1H), 7.80 (m, 1H), 7.38 (m, 1H), 7.10–7.05 (m, 1H), 6.85–6.75 (m, 1H), 6.45–6.38 (m, 2H), 5.62 (m, 1H), 3.98 (m, 2H), 3.60 (m, 2H), 3.50 (m, 2H), 3.32 (m, 1H), 2.98–2.85 (m, 2H), 1.20 (m, 3H). Mass spectrum, ESI-MS *m/z* 543 $(M + H)^+$, calc'd for $C_{24}H_{25}BFN_4O_9$.

Compound 6: (3*R*)-3-(2-(2,3-difluoro-4-hydroxyphenyl)-2-(4-ethyl-2,3-dioxopiperazine-1-carboxamido)acetamido)-2-hydroxy-3,4-dihydro-2H-benzo[e][1,2]oxaborinine-8-carboxylic acid. ^{1}H NMR (MeOH-d_4): δ 9.59–9.38 (m, 1H), 7.88–7.68 (m, 1H), 7.36–7.14 (m, 1H), 7.04–6.83 (m, 1H), 6.61–6.31 (m, 2H), 5.70–5.62 (m, 1H), 4.05–3.93 (m, 2H), 3.70–3.61 (m, 2H), 3.56–3.49 (m, 2H), 3.36 (s, 1H), 3.01–2.84 (m, 2H), 1.22–1.18 (m, 3H). Mass spectrum, ESI-MS *m/z* 561 (M + H)$^+$, calc'd for $C_{24}H_{24}BF_2N_4O_9$.

Compound 7: (3*R*)-3-(2-(3,5-difluoro-4-hydroxyphenyl)-2-(4-ethyl-2,3-dioxopiperazine-1-carboxamido)acetamido)-2-hydroxy-3,4-dihydro-2H-benzo[e][1,2]oxaborinine-8-carboxylic acid. ^{1}H NMR (MeOH-d_4): δ 9.57–9.30 (m, 1H), 7.76 (dd, *J* = 10.8, 4.5 Hz, 1H), 7.32–7.09 (m, 1H), 7.00–6.80 (m, 1H), 6.62–6.59 (m, 2H), 5.36–5.33 (m, 1H), 4.05–3.93 (m, 2H), 3.69–3.59 (m, 2H), 3.56–3.47 (m, 2H), 3.39–3.31 (m, 1H), 3.00–2.78 (m, 2H), 1.25–1.16 (m, 3H). Mass spectrum, ESI-MS *m/z* 561 (M + H)$^+$, calc'd for $C_{24}H_{24}BF_2N_4O_9$.

Compound 8: (3*R*)-3-(2-(2,5-difluoro-4-hydroxyphenyl)-2-(4-ethyl-2,3-dioxopiperazine-1-carboxamido)acetamido)-2-hydroxy-3,4-dihydro-2H-benzo[e][1,2]oxaborinine-8-carboxylic acid. ^{1}H NMR (MeOH-d_4): δ 9.62–9.41 (m, 1H), 7.85–7.77 (m, 1H), 7.36–7.16 (m, 1H), 7.04–6.85 (m, 1H), 6.79–6.63 (m, 1H), 6.61–6.49 (m, 1H), 5.71–5.62 (m, 1H), 4.07–3.96 (m, 2H), 3.68–3.65 (m, 2H), 3.58–3.50 (m, 2H), 3.38–3.37 (m, 1H), 3.00 (d, *J* = 2.8 Hz, 1H), 2.94–2.86 (m, 1H), 1.25–1.20 (m, 3H). Mass spectrum, ESI-MS *m/z* 561 (M + H)$^+$, calc'd for $C_{24}H_{24}BF_2N_4O_9$.

Compound 9: (3*R*)-3-(2-(4-ethyl-2,3-dioxopiperazine-1-carboxamido)-2-(2,3,5-trifluoro-4-hydroxyphenyl)acetamido)-2-hydroxy-3,4-dihydro-2H-benzo[e][1,2]oxaborinine-8-carboxylic acid. ^{1}H NMR (MeOH-d_4): δ 9.67–9.45 (m, 1H), 7.77–7.73 (m, 1H), 7.33–7.14 (m, 1H), 7.01–6.83 (m, 1H), 6.71–6.39 (m, 1H), 5.71–5.68 (m, 1H), 4.00–3.98 (m, 2H), 3.66–3.62 (m,

2H), 3.58–3.46 (m, 2H), 3.36–3.32 (m, 1H), 3.01–2.81 (m, 2H), 1.21–1.18 (m, 3H). Mass spectrum, ESI-MS *m/z* 579 $(M + H)^+$, calculated for $C_{24}H_{23}BF_3N_4O_9$.

Compound 10: The preparation of (3*R*)-3-(2-(4-carboxyphenyl)-2-(4-ethyl-2,3-dioxopiperazine-1-carboxamido)-acetamido)-2-hydroxy-3,4-dihydro-2H-benzo[e][1,2]oxaborinine-8-carboxylic acid:

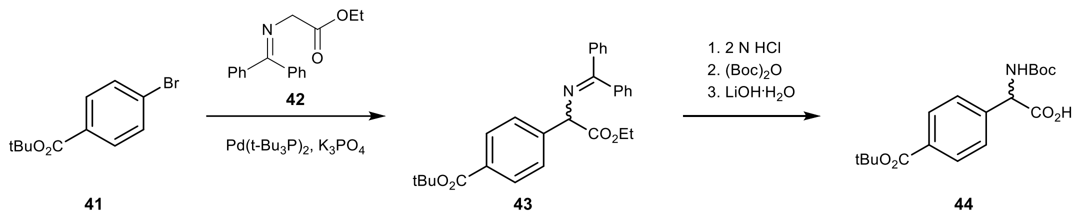

Scheme S5. The synthesis of racemic, benzoate-containing aryl glycine **42**.

Step 1: ***tert*-Butyl 4-(1-((diphenylmethylene)amino)-2-ethoxy-2-oxoethyl)benzoate (43)**. To a solution of *tert*-butyl 4-bromo-benzoate (**41**, 7 g, 27 mmol) in toluene (80 mL), $Pd(t\text{-}Bu_3P)_2$ (2 g, 3.9 mmol), K_3PO_4 (17 g, 81 mmol), and ethyl *N*-(diphenylmethylene) glycinate (**42**, 10 g, 38 mmol) were added at ambient temperature, and the reaction mixture was warmed to 145 °C under an argon atmosphere. After 18 h, the reaction mixture was concentrated to dryness and the crude residue was purified by flash silica gel chromatography (EtOAc/petroleum ether, 12:1) to afford **43** (1.8 g, 15%) as a brown-colored oil. Mass spectrum, ESI-MS m/z: 444 $(M + H)^+$, calculated for $C_{28}H_{30}NO_4$.

Step 2: **2-((*tert*-Butoxycarbonyl)amino)-2-(4-(*tert*-butoxycarbonyl)phenyl)acetic acid (44)**. A solution of **43** (2.3 g, 5 mmol) in 2N HCl in diethyl ether (20 mL) was maintained at ambient temperature. After 4 h, water (1 mL) was added to the reaction mixture. After 2 min, the reaction mixture was dried over anhydrous Na_2SO_4 and filtered. The filtrate was diluted with EtOAc (60 mL) and then concentrated to provide the crude HCl salt (2.4 g) as a brown oil. Mass spectrum, ESI-MS *m/z* 280 $(M + H)^+$, calculated for $C_{15}H_{22}NO_4$. To the crude HCl salt (2.4 g, 8 mmol) in THF (20 mL), DIPEA (3 mL, 17 mmol) and Boc2O (3.5 g, 16 mmol) were added, and the reaction

mixture was maintained at ambient temperature. After 18 h, the reaction mixture was concentrated to dryness, and the crude residue was purified by flash silica gel chromatography (20:1 petroleum ether/EtOAc) to obtain the *N*-Boc-protected ester as a yellow-colored oil (1.6 g, 49%). Mass spectrum, ESI-MS *m/z* 380 $(M + H)^+$, calculated for $C_{20}H_{30}NO_6$. To the *N*-Boc-protected ester (1.6 g, 4 mmol) in THF (15 mL), 2N LiOH (15 mL, 30 mmol) was added, and the reaction mixture was stirred at ambient temperature. After 4 h, the reaction mixture was acidified to pH 3 with aqueous HCl, extracted with EtOAc (3 × 50 mL), dried over anhydrous Na_2SO_4, filtered, and concentrated. The crude acid was purified by flash silica gel chromatography (1:1 EtOAc/petroleum ether) to obtain **44** (1 g, 67%) as a white-colored solid. Mass spectrum, ESI-MS *m/z* 352 $(M + H)^+$, calculated for $C_{18}H_{26}NO_6$.

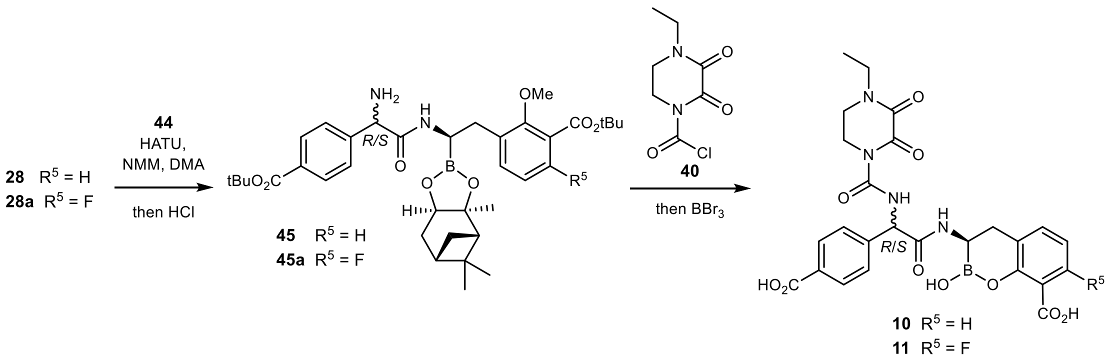

Scheme S6. Preparation of benzoate-based boro-PBPi **10** and **11**.

Step 3: **(3*R*)-3-(2-(4-carboxyphenyl)-2-(4-ethyl-2,3-dioxopiperazine-1-carboxamido)-acetamido)-2-hydroxy-3,4-dihydro-2H-benzo[e][1,2]oxaborinine-8-carboxylic acid (10).** Prepared using the general methods described for the preparation of **2**. ^{1}H NMR (MeOH-*d₄*): δ 9.62–9.40 (m, 1H), 7.85–7.70 (m, 3H), 7.35 (m, 1H), 7.05 (m, 2H), 6.78 (m, 1H), 5.59 (m, 1H), 4.00 (m, 2H), 3.62 (m, 2H), 3.50 (m, 2H), 3.32 (s, 1H), 2.99 -2.82 (m, 2H), 1.20 (m, 3H). Mass spectrum, ESI-MS *m/z* 553 $(M + H)^+$, calculated for $C_{25}H_{26}BN_4O_{10}$.

Boro-PBPi **11–14** were prepared following the general methods outlined for the synthesis of **10**.

Compound 11: (3*R*)-3-(2-(4-carboxyphenyl)-2-(4-ethyl-2,3-dioxopiperazine-1-carboxamido)acetamido)-7-fluoro-2-hydroxy-3,4-dihydro-2H-benzo[e][1,2]oxaborinine-8-carboxylic acid. ^{1}H NMR (MeOH-d_4): δ 9.70–9.42 (m, 1H), 7.99–7.82 (m, 2H), 7.25–7.10 (m, 2H), 6.75–6.62 (m, 1H), 6.30 (m, 1H), 5.58 (m, 1H), 4.00 (m, 2H), 3.62 (m, 2H), 3.50 (m, 2H), 3.20 (s, 1H), 2.80 (m, 2H), 1.20 (m, 3H). Mass spectrum, ESI-MS *m/z* 571 (M + H)$^+$, calculated for $C_{25}H_{25}BFN_4O_{10}$.

Compound 12: (*R*)-3-((*R*)-2-(4-carboxyphenyl)-2-(4-ethyl-2,3-dioxopiperazine-1-carboxamido)acetamido)-2-hydroxy-3,4-dihydro-2H-benzo[e][1,2]oxaborinine-8-carboxylic acid. ^{1}H NMR (MeOH-d_4): δ 9.69 (d, *J* = 5.5 Hz, 1H), 7.81 (d, *J* = 8.3 Hz, 2H), 7.72 (dd, *J* = 7.9, 1.2 Hz, 1H), 7.19–7.07 (m, 3H), 6.79 (t, *J* = 7.6 Hz, 1H), 5.69–5.55 (m, 1H), 4.04–4.01 (m, 2H), 3.74–3.61 (m, 2H), 3.54 (q, *J* = 7.2 Hz, 2H), 3.43–3.38 (m, 1H), 2.95–2.83 (m, 2H), 1.22 (t, *J* = 7.2 Hz, 3H). Mass spectrum, ESI-MS *m/z* 553 (M + H)$^+$, calc'd for $C_{25}H_{26}BN_4O_{10}$.

Compound 13: (*R*)-3-((*S*)-2-(4-carboxy-3-fluorophenyl)-2-(4-ethyl-2,3-dioxopiperazine-1-carboxamido)acetamido)-2-hydroxy-3,4-dihydro-2H-benzo[e][1,2]oxaborinine-8-carboxylic acid. ^{1}H NMR (MeOH-d_4): δ 7.84–7.74 (m, 2H), 7.34 (d, *J* = 5.9 Hz, 1H), 7.02 (t, *J* = 7.6 Hz, 1H), 6.94 (dd, *J* = 10.9, 1.5 Hz, 1H), 6.85 (dd, *J* = 8.2, 1.4 Hz, 1H), 5.62 (s, 1H), 4.01 (t, *J* = 5.8 Hz, 2H), 3.70–3.68 (m, 2H), 3.60–3.52 (m, 3H), 3.00 (d, *J* = 3.1 Hz, 2H), 1.24 (t, *J* = 7.2 Hz, 3H). Mass spectrum, ESI-MS *m/z* 571 (M + H)$^+$, calc'd for $C_{25}H_{25}BFN_4O_{10}$.

Compound 14: (*R*)-3-((*R*)-2-(4-carboxy-2,3-difluorophenyl)-2-(4-ethyl-2,3-dioxopiperazine-1-carboxamido)acetamido)-2-hydroxy-3,4-dihydro-2H-benzo[e][1,2]oxaborinine-8-carboxylic acid. ^{1}H NMR (MeOH-d_4): δ 7.76 (dd, *J* = 7.9, 1.7 Hz, 1H), 7.60–7.50 (m, 1H), 7.34 (dd, *J* = 7.3, 1.5 Hz, 1H), 7.02 (t, *J* = 7.6 Hz, 1H), 6.74 (t, *J* = 7.0 Hz, 1H), 5.90 (d, *J* = 1.3 Hz, 1H), 4.02–3.99 (m, 2H), 3.65 (t, *J* = 5.7 Hz, 2H), 3.56–3.50 (m, 2H), 3.36–3.32 (m, 1H), 2.98 (d, *J* =

3.1 Hz, 2H), 1.21 (t, *J* = 7.2 Hz, 3H). Mass spectrum, ESI-MS *m/z* 589 $(M + H)^+$, calculated for $C_{25}H_{24}BF_2N_4O_{10}$.

Compound 15: The preparation of (*R*)-3-((*R*)-2-(4-ethyl-2,3-dioxopiperazine-1-carboxamido)-2-(4-phosphonophenyl)acetamido)-2-hydroxy-3,4-dihydro-2H-benzo[e][1,2]oxaborinine-8-carboxylic acid:

Step 1: **2-Amino-2-(4-iodophenyl)acetonitrile (33)**. To a solution of benzaldehyde **31** (Scheme 3, 1 g, 4 mmol) in methanolic ammonia (7 N NH_3/methanol, 23 mL) at 0 °C, trimethylsilyl cyanide (0.75 mL, 6 mmol) was added, and the reaction mixture was warmed to 45 °C. After 7 h, the reaction mixture was concentrated to yield crude **33**, which was used without further purification.

Step 2: **Methyl 2-((*tert*-butoxycarbonyl)amino)-2-(4-iodophenyl)acetate (35)**. Crude **33** (4 mmol) was dissolved in 3 N HCl in MeOH (14 mL) and warmed to 70 °C. After 18 h, the reaction mixture was concentrated and used without purification. The crude residue was suspended in THF (20 mL) and cooled to 0 °C. Triethylamine (1.8 mL, 12.0 mmol) was added, followed by di-*tert*-butyl dicarbonate (1.4 g, 6.0 mmol), and the reaction mixture was warmed to ambient temperature. After 1 h, the reaction mixture was concentrated to dryness. The crude product was purified by flash silica gel chromatography (10% EtOAc/hexanes) to yield compound **35** as a light tan-colored solid (1.0 g, 85% yield). Mass spectrum, ESI-MS *m/z* 392 $(M + H)^+$, calculated for $C_{14}H_{19}INO_4$.

Step 3: **2-((*tert*-Butoxycarbonyl)amino)-2-(4-iodophenyl)acetic acid (37)**. To a solution of **35** (1.1 g, 2.8 mmol) in 1:1 THF/water (20 mL), LiOH hydrate (0.35 g, 8 mmol) was added, and the reaction mixture was maintained at ambient temperature. After 1 h, the reaction mixture was concentrated, adjusted to pH 2 with 2 N HCl, and extracted with DCM. The combined organic extracts were washed with water, dried over anhydrous Na_2SO_4, filtered, and concentrated to yield **37** (0.9 g, 94% yield) as a white-colored solid. Mass spectrum, ESI-MS *m/z* 378 $(M + H)^+$, calculated for $C_{13}H_{17}INO_4$.

Step 4: ***tert*-Butyl 3-((2*R*)-2-(2-amino-2-(4-iodophenyl)acetamido)-2-((3a*S*,4*S*,6*S*,7a*R*)-3a,5,5-trimethylhexahydro-4,6-methanobenzo[d][1,3,2]dioxaborol-2-yl)ethyl)-2-methoxybenzoate (39)**. To a solution of **27** (0.7 g, 1.6 mmol) in THF (6 mL) at −78 °C, a solution of lithium hexamethyldisilazide (1 M LHMDS/THF, 1.6 mL, 1.6 mmol) was added dropwise. Following the addition, the −78 °C bath was removed, and the reaction mixture was slowly warmed to ambient temperature. After 18 h, the resulting solution of **28** (*ca.* 1.6 mmol) in THF was used without further purification.

To a mixture of **37** (1.1 g, 3.1 mmol) and HATU (1.4 g, 3.6 mmol) in DMA (6 mL) was added NMM (0.44 mL, 4 mmol) at ambient temperature. After 90 min, the previously prepared solution of **28** (*ca.* 1.6 mmol) in THF was added. After 2.5 h, the reaction mixture was diluted with EtOAc, washed successively with water and brine, dried over anhydrous Na_2SO_4, filtered, and concentrated. The crude residue was purified by flash silica gel chromatography (30% EtOAc/hexanes) to provide the coupled product as an off-white-colored solid (0.75 g, 61% yield). Mass spectrum, ESI-MS *m/z* 789 $(M + H)^+$, calculated for $C_{37}H_{51}BN_2O_8$.

The coupled product (0.75 g, 0.95 mmol) was added to 2 N HCl in diethyl ether (17 mL, 33 mmol) at 0 °C, and the reaction mixture was warmed to ambient temperature. After 18 h, the reaction mixture was concentrated to give **39** as a tan-colored solid, which was used without further purification (0.69 g, quantitative yield). Mass spectrum, ESI-MS *m/z* 689 $(M + H)^+$, calculated for $C_{32}H_{43}BN_2O_6$.

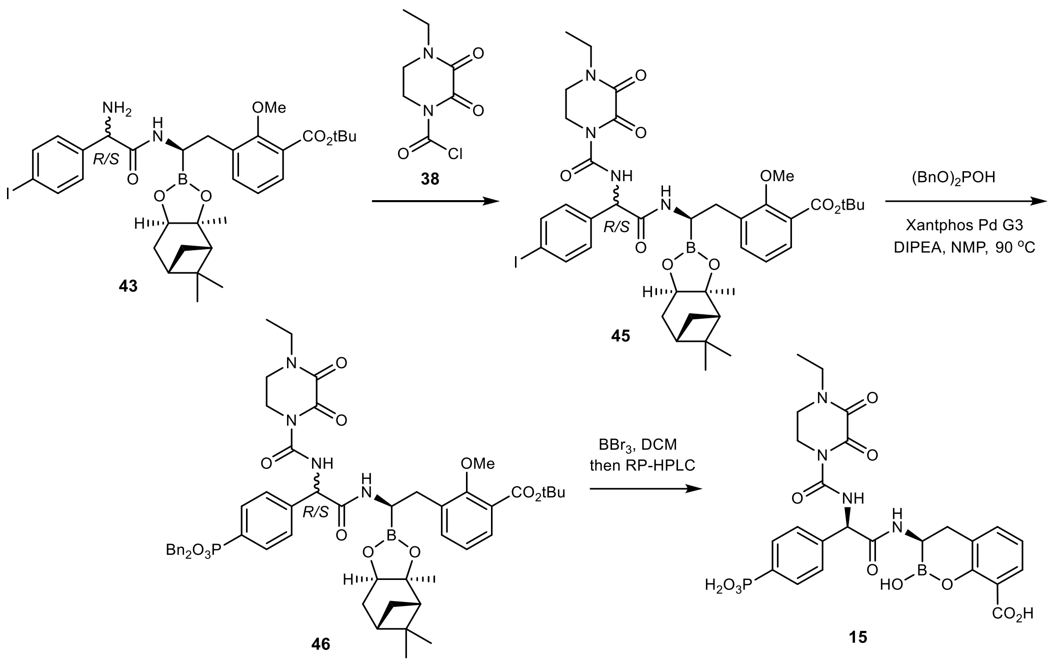

Scheme S7. Preparation of phosphonate-containing boro-PBPi **15**.

Step 5: ***tert*-Butyl 3-((2*R*)-2-(2-(4-ethyl-2,3-dioxopiperazine-1-carboxamido)-2-(4-iodophenyl)acetamido)-2-((3a*S*,4*S*,6*S*,7a*R*)-3a,5,5-trimethylhexahydro-4,6-methanobenzo[d]-[1,3,2]dioxaborol-2-yl)ethyl)-2-methoxybenzoate (46).** A solution of crude **39** (0.69 g, 0.9 mmol) in DCM (16 mL) was cooled to 0 °C. DIPEA (0.5 mL, 2.8 mmol) was added, followed by **40** (0.3 g, 1.4 mmol), and the reaction mixture was warmed to ambient temperature. After 0.5 h, the reaction mixture was poured into water and extracted with DCM. The combined organic extracts were dried over anhydrous Na_2SO_4, filtered, and concentrated to give ureido intermediate **46,** which was used without further purification. Mass spectrum, ESI-MS *m/z*: 857 $(M + H)^+$, calculated for $C_{39}H_{51}BIN_4O_9$.

Step 6: ***tert*-Butyl 3-((2*R*)-2-(2-(4-((dibenzyl-l³-oxidaneyl)(l¹-oxidaneyl)phosphoryl)phenyl)-2-(4-ethyl-2,3-dioxopiperazine-1-carboxamido)acetamido)-2-((3a*S*,4*S*,6*S*,7a*R*)-3a,5,5-trimethylhexahydro-4,6-methanobenzo[d][1,3,2]dioxaborol-2-yl)ethyl)-2-methoxybenzoate (47)**. To a solution of **46** (0.25 g, 0.3 mmol) in NMP (6 mL) was added DIPEA (0.15 mL, 0.95 mmol), [(4,5-bis(diphenylphosphino)-9,9-dimethylxanthene)-2-(2′-amino-1,1′-

biphenyl)]palladium(II) methanesulfonate (Xantphos Pd G3, 0.03 g, 0.03 mmol), and dibenzyl phosphite (0.14 mL, 0.63 mmol). The reaction mixture was degassed (three times) under argon and then warmed to 90 °C. After 1 h, the reaction mixture was cooled, diluted with EtOAc, washed with water, dried over anhydrous Na_2SO_4, filtered, and concentrated to obtain **47** as an oil, which was used without further purification. Mass spectrum, ESI-MS *m/z* 991 $(M + H)^+$, calculated for $C_{53}H_{65}BN_4O_{12}P$.

Step 7: **(*R*)-3-((*R*)-2-(4-ethyl-2,3-dioxopiperazine-1-carboxamido)-2-(4-phosphonophenyl)-acetamido)-2-hydroxy-3,4-dihydro-2H-benzo[e][1,2]oxaborinine-8-carboxylic acid (15)**. To a solution of **47** (0.28 g, 0.3 mmol) in anhydrous DCM (8 mL) at -78 °C was slowly added BBr_3 (1.0 M in DCM, 2.8 mL, 2.8 mmol) and the reaction mixture was slowly warmed to 0 °C. After 2 h, the reaction mixture was quenched with water (2 mL) and methanol (5 mL), concentrated to remove DCM, and purified by preparative RP-HPLC [Waters XBridge™ C18 column (5-45% ACN in water containing 0.1% TFA over 10 min; Flow rate: 45 mL/min. Peak 1: *S*,*R*-diastereomer (undesired), RT = 1.71 min; Peak 2: *R*,*R*-diastereomer (desired), RT = 1.79 min]. The product-containing fractions were combined and lyophilized to dryness to provide **15** as a white-colored solid (30 mg, 20% yield). ^{1}H NMR (MeOH-d_4): δ 9.36 (d, *J* = 4.4 Hz, 1H), 7.83 (d, *J* = 8.0 Hz, 1H), 7.68 (m, 2H), 7.35 (d, *J* = 7.1 Hz, 1H), 7.08 (m, 1H), 6.96 (m, 2H), 5.56 (d, *J* = 3.5 Hz, 1H), 3.95 (m, 2H), 3.65 (m, 2H), 3.53 (m, 2H), 3.30 (m, 1H), 2.99 (s, 2H), 1.21 (t, *J* = 7.2 Hz, 3H). Mass spectrum, ESI-MS *m/z* 589 $(M + H)^+$, calculated for $C_{24}H_{27}BN_4O_{11}P$.

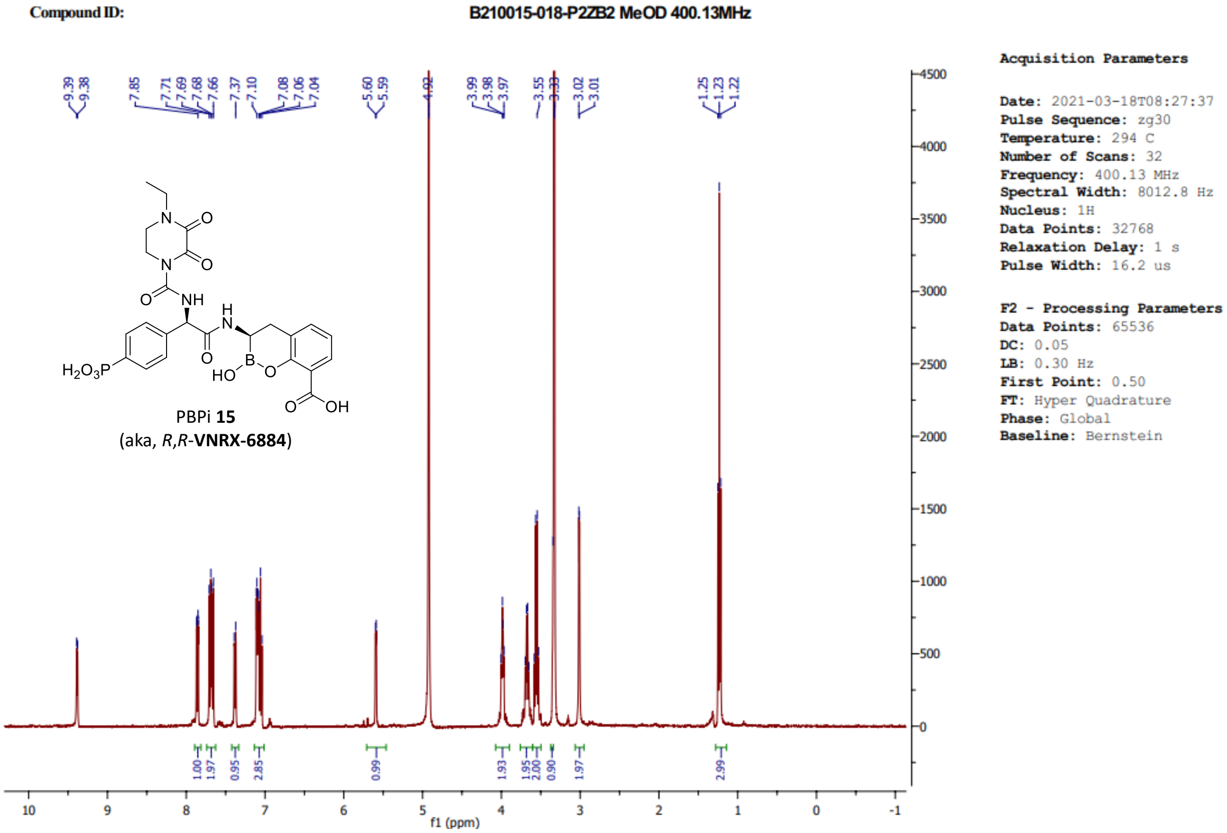

Figure S8. The ^{1}H NMR spectrum of boro-PBPi **15** in MeOH-d_4.

```
Print of window 79: MS Spectrum
Data File  : C:\USERS\ZHOUX2\DESKTOP\光化学讲座\VNRX-6884\P2E\B210015-018-P2ZB2-51405.D
Sample Name : B210015-018-P2ZB2
=====================================================================
Acq. Operator   : sysadmin
Acq. Instrument : Agilent LCMS A                Location : P1-A-01
Injection Date  : 3/18/2021 7:48:51 AM               Inj :    1
                                             Inj Volume : 1.000 µl
Acq. Method     : D:\DATA\1\METHODS\P100-1000.M
Last changed    : 11/27/2019 10:24:05 AM by sysadmin
Analysis Method : C:\CHEM32\1\METHODS\DEF_LC.M
Last changed    : 11/20/2006 6:14:44 PM
```

MS Spectrum

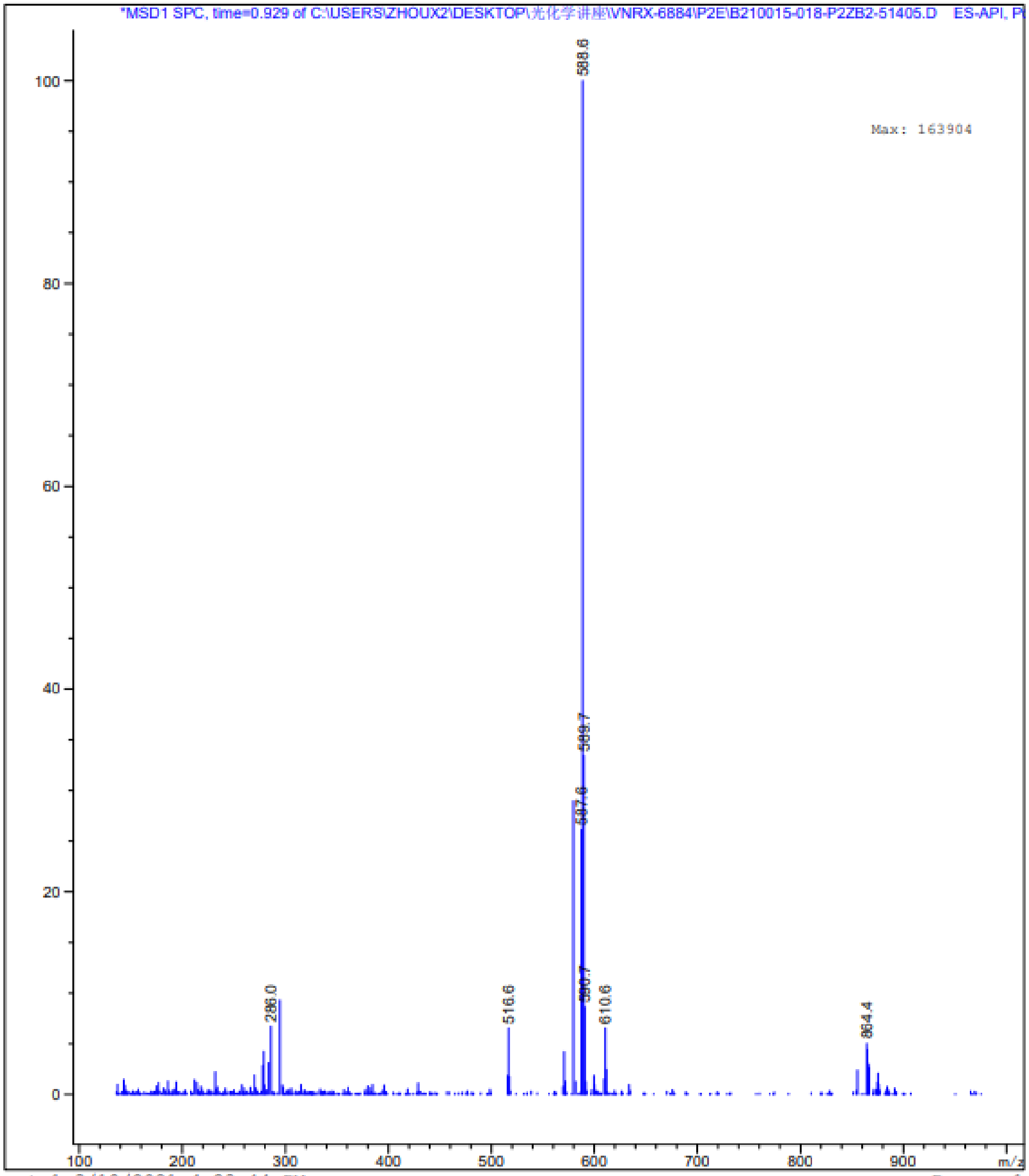

Figure S9. Mass spectral data for boro-PBPi **15**.

Boro-PBPi **16** and **17** were prepared following the general methods outlined for the synthesis of **15**.

Compound 16: (*R*)-3-((*R*)-2-(4-ethyl-2,3-dioxopiperazine-1-carboxamido)-2-(3-fluoro-4-phosphonophenyl)acetamido)-2-hydroxy-3,4-dihydro-2H-benzo[e][1,2]oxaborinine-8-carboxylic acid. ^{1}H NMR (MeOH-d_4): δ 9.36 (d, *J* = 4.4 Hz, 1H), 7.87 (d, *J* = 2.8 Hz, 1H), 7.65 (m, 1H), 7.35 (m, 1H), 7.08 (m, 1H), 6.96 (m, 1H), 6.75 (m, 1H), 5.56 (d, *J* = 3.5 Hz, 1H), 3.95 (m, 2H), 3.65 (m, 2H), 3.53 (m, 2H), 3.30 (m, 1H), 2.99 (s, 2H), 1.21 (t, *J* = 7.2 Hz, 3H). Mass spectrum, ESI-MS *m/z* 607 $(M + H)^+$, calculated for $C_{24}H_{26}BFN_4O_{11}P$.

Compound 17: (*R*)-3-((*R*)-2-(4-ethyl-2,3-dioxopiperazine-1-carboxamido)-2-(4-phosphonophenyl)-acetamido)-7-fluoro-2-hydroxy-3,4-dihydro-2H-benzo[e][1,2]oxaborinine-8-carboxylic acid. ^{1}H NMR (MeOH-d_4): δ 9.44 (m, 1H), 7.76 (m, 2H), 7.27 (m, 2H), 7.12 (m, 1H), 6.67 (m, 1H), 5.55 (m, 1H), 4.08 (m, 1H), 3.87 (m, 1H), 3.78 (m, 1H), 3.58 (m, 3H), 3.30 (m, 1H), 2.87 (s, 2H), 1.21 (t, *J* = 7.2 Hz, 3H). Mass spectrum, ESI-MS *m/z* 607 $(M + H)^+$, calc'd for $C_{24}H_{26}BFN_4O_{11}P$.

Compound 18: The preparation of (*R*)-2-hydroxy-3-((*R*)-2-(3-(methylsulfonyl)-2-oxoimidazolidine-1-carboxamido)-2-(4-phosphonophenyl)acetamido)-3,4-dihydro-2H-benzo[e][1,2]oxaborinine-8-carboxylic acid:

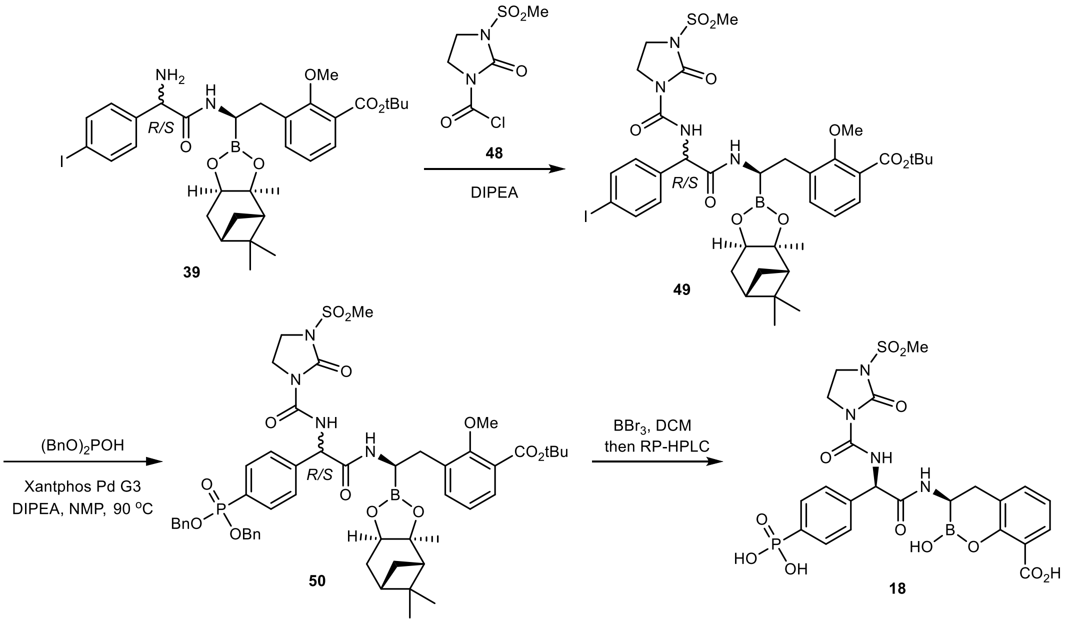

Scheme S8. Preparation of phosphonate-containing boro-PBPi **18**.

Step 1: ***tert*-Butyl 3-((2*R*)-2-(2-(4-iodophenyl)-2-(3-(methylsulfonyl)-2-oxoimidazolidine-1-carboxamido)acetamido)-2-((3a*S*,4*S*,6*S*,7a*R*)-3a,5,5-trimethylhexahydro-4,6-methano-benzo[d][1,3,2]dioxaborol-2-yl)ethyl)-2-methoxybenzoate (49).** Synthesized using the general method for the preparation of **15** but utilizing 3-(methylsulfonyl)-2-oxoimidazolidine-1-carbonyl chloride (**48**) in place of **40**. Crude **49** was used without further purification. Mass spectrum, ESI-MS *m/z* 879 (M + H)$^+$, calculated for $C_{37}H_{49}BIN_4O_{10}S$.

Step 2: ***tert*-Butyl 3-((2*R*)-2-(2-(4-((dibenzyl-l^3-oxidaneyl)(l^1-oxidaneyl)phosphoryl)phenyl)-2-(3-(methylsulfonyl)-2-oxoimidazolidine-1-carboxamido)acetamido)-2-((3a*S*,4*S*,6*S*,7a*R*)-3a,5,5-trimethylhexahydro-4,6-methanobenzo[d][1,3,2]dioxaborol-2-yl)ethyl)-2-methoxybenzoate (50)**. The compound was synthesized using the general method described for the preparation of **15**. Crude **50** was used without further purification. Mass spectrum, ESI-MS *m/z* 1013 (M + H)$^+$, calculated for $C_{51}H_{63}BIN_4O_{13}PS$.

Step 3: **(*R*)-2-Hydroxy-3-((*R*)-2-(3-(methylsulfonyl)-2-oxoimidazolidine-1-carboxamido)-2-(4-phosphonophenyl)acetamido)-3,4-dihydro-2H-benzo[e][1,2]oxaborinine-8-carboxylic acid (18)**. The compound was synthesized and purified using the general methods described for the preparation of **15**. ^{1}H NMR (MeOH-d_4): δ 8.38 (m, 1H), 7.97 (d, J = 10.6 Hz, 1H), 7.66 (m, 2H), 7.36 (m, 1H), 7.05 (m, 3H), 5.59 (m, 1H), 3.91 (m, 2H), 3.72 (m, 2H), 3.34 (m, 1H), 3.30 (s, 3H), 2.99 (s, 2H). Mass spectrum, ESI-MS m/z 611 (M + H)$^+$, calculated for $C_{22}H_{25}BN_4O_{12}PS$.

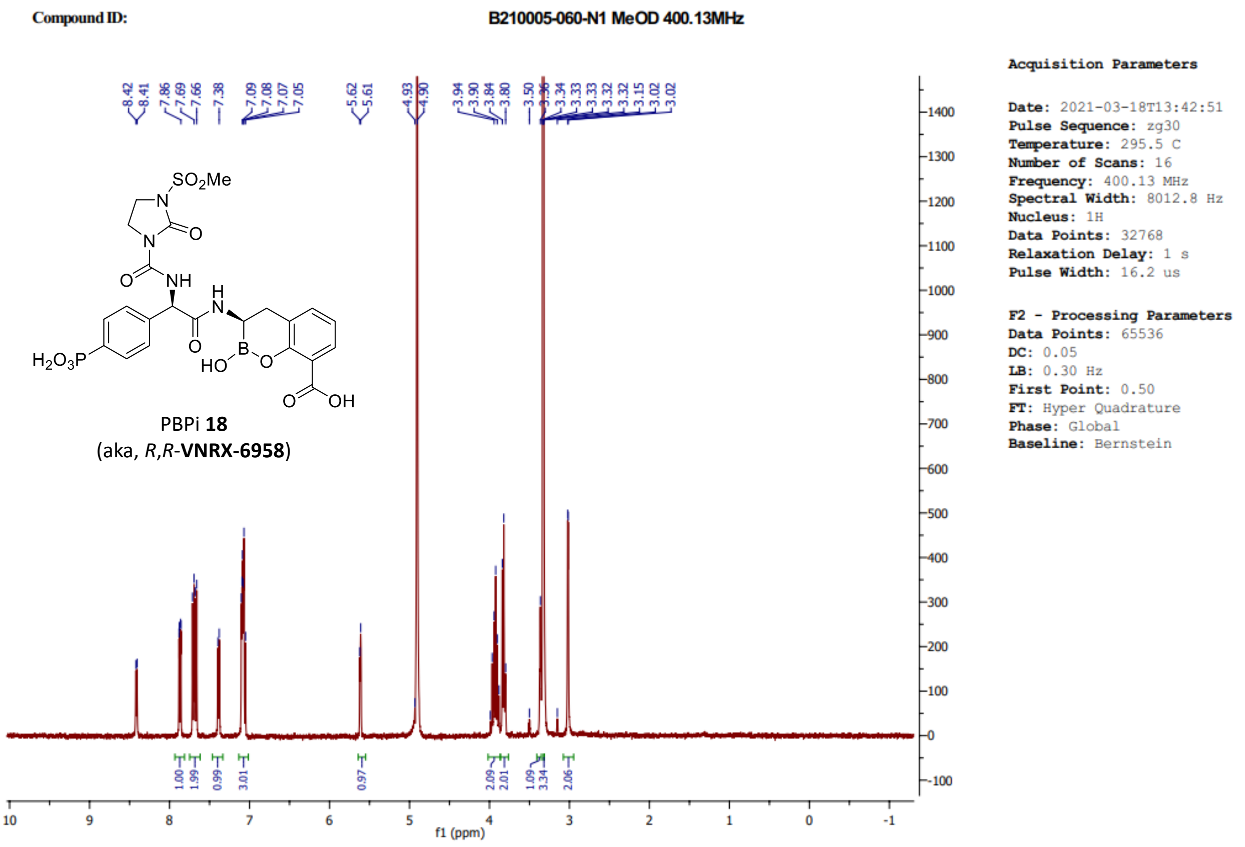

Figure S10. The ^{1}H NMR spectrum of boro-PBPi **18** in MeOH-d_4.

```
Print of window 79: MS Spectrum
Data File   : C:\USERS\ZHOUX2\DESKTOP\光化学讲座\VNRX-6958-P2\B210005-060-PP2-01878.D
Sample Name : B210005-060-PP2
======================================================================
Acq. Operator    : admin
Acq. Instrument : LCMS-C                          Location : P1-D-07
Injection Date   : 3/18/2021 1:39:08 PM                 Inj :    1
                                                Inj Volume : 20.000 µl
Different Inj Volume from Sequence !     Actual Inj Volume : 1.000 µl
Acq. Method      : D:\CHEMSTATION\1\METHODS\P100-1000.M
Last changed     : 12/21/2020 1:00:41 PM by admin
Analysis Method : C:\CHEM32\1\METHODS\DEF_LC.M
Last changed     : 11/20/2006 6:14:44 PM
```

MS Spectrum

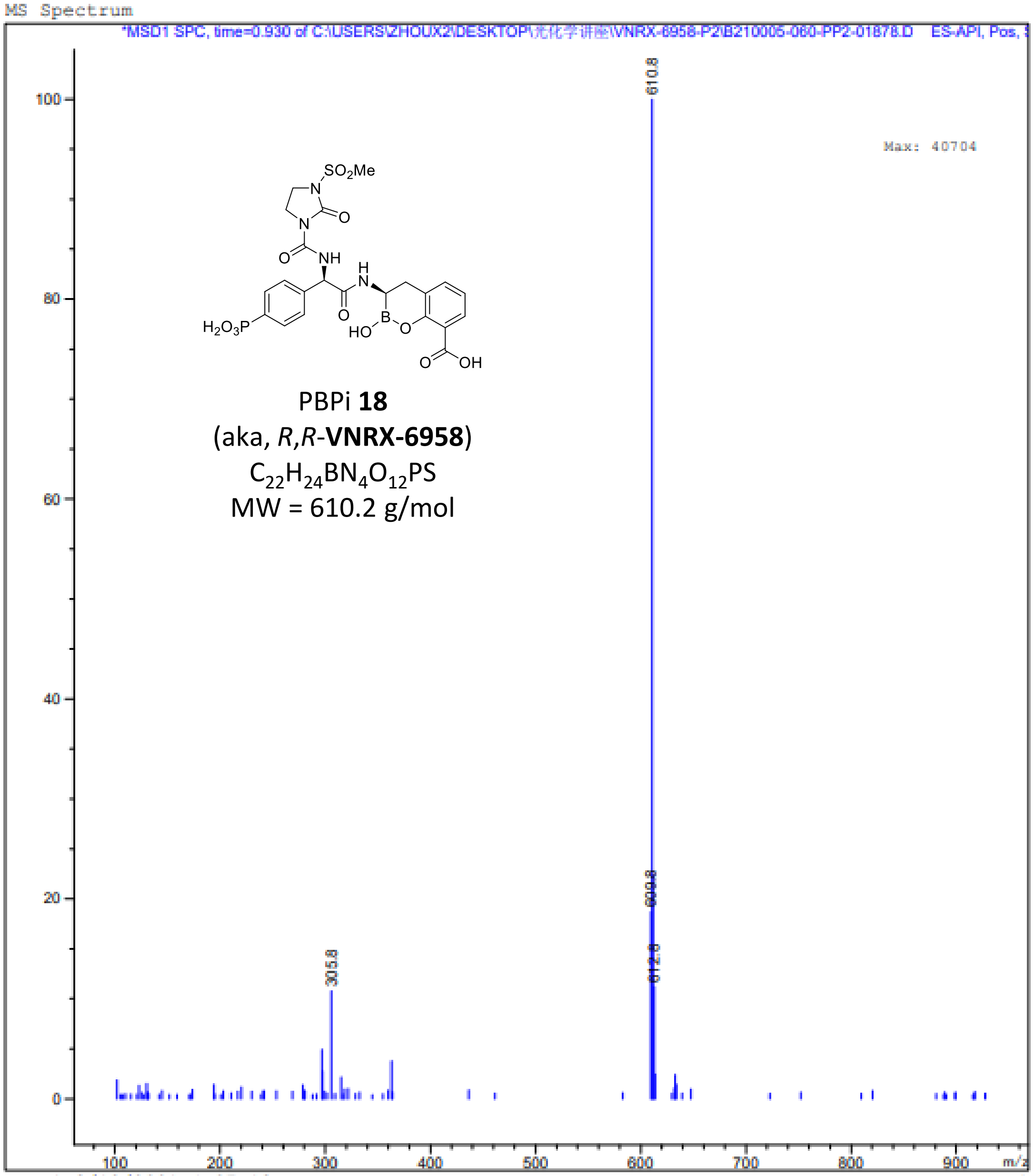


```
Instrument 1 3/18/2021 4:27:13 PM                                    Page    1 of 1
```

Figure S11. Mass spectral data for boro-PBPi **18**.

Compound 21 (VNRX-14079): The preparation of (*R*)-7-fluoro-3-((*R*)-2-(3-fluoro-4-phosphonophenyl)-2-(3-(methylsulfonyl)-2-oxoimidazolidine-1-carboxamido)acetamido)-2-hydroxy-3,4-dihydro-2H-benzo[e][1,2]oxaborinine-8-carboxylic acid:

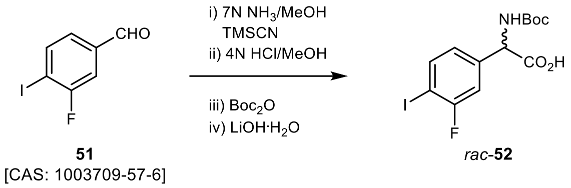

Scheme S9. Preparation of 3-fluoro-4-iodo phenyl glycinate **52**.

Step 1: To 3-fluoro-4-iodobenzaldehyde (**51**, 2 g, 8 mmol) at 0 °C, 7N NH_3 in MeOH (43 mL) was added, followed by trimethylsilyl cyanide (1.5 mL, 12 mmol, 1.5 eq), and the reaction mixture was warmed to 45 °C. After 7 h, the reaction mixture was concentrated *in vacuo* to provide crude *rac*-2-amino-2-(3-fluoro-4-iodophenyl)acetonitrile (2.2 g, quant.) which was used in the next step without further purification. Mass spectrum, *m/z* (ESI) 277 $(M + H)^+$, calculated for $C_8H_7FIN_2$.

Step 2: Crude 2-amino-2-(3-fluoro-4-iodophenyl)acetonitrile (2.2 g, 8 mmol) was dissolved in HCl/dioxane (4 N, 28 mL) and MeOH (28 mL) and warmed to 70 °C. After 18 h, the homogeneous reaction mixture was concentrated to provide crude *rac*-methyl 2-amino-2-(3-fluoro-4-iodophenyl)acetate hydrogen chloride (2.8 g, quant.) which was used in the next step without further purification. Mass spectrum, *m/z* (ESI) 310 $(M + H)^+$, calculated for $C_9H_{10}FINO_2$.

Step 3: Crude methyl 2-amino-2-(3-fluoro-4-iodophenyl)acetate hydrogen chloride (2.8 g, 8 mmol) was suspended in THF (30 mL) and cooled to 0 °C. Triethylamine (3.3 mL, 24 mmol, 3 eq) was added followed by di-*tert*-butyl dicarbonate (2.6 g, 12 mmol, 1.5 eq) and the reaction mixture was warmed to ambient temperature. After 1 h, the reaction mixture was concentrated, and the crude product was purified by flash silica gel chromatography (10% EtOAc/hexanes) to provide *rac*-methyl 2-((*tert*-butoxycarbonyl)amino)-2-(3-fluoro-4-iodophenyl)acetate (1.9 g, 57%) as an off-white-colored solid. Mass spectrum, *m/z* (ESI) 410 $(M + H)^+$, calculated for $C_{14}H_{18}FINO_4$.

Step 4: To a solution of methyl 2-((*tert*-butoxycarbonyl)amino)-2-(3-fluoro-4-iodophenyl)acetate (1.9 g, 4.6 mmol) in 1:1 THF/H_2O (36 mL) was added lithium hydroxide monohydrate (0.57 g, 13.6 mmol, 3 eq) at ambient temperature. After 1 h, the homogeneous solution was concentrated to remove THF. The aqueous solution was made acidic (pH 2) by the dropwise addition of 2N HCl and extracted with CH_2Cl_2. The organic extract was washed with water, dried over anhydrous Na_2SO_4, filtered, and concentrated to afford *rac*-**52** (1.8 g, quant.) as an off-white-colored solid. Mass spectrum, *m/z* (ESI) 396 $(M + H)^+$, calculated for $C_{13}H_{16}FINO_4$.

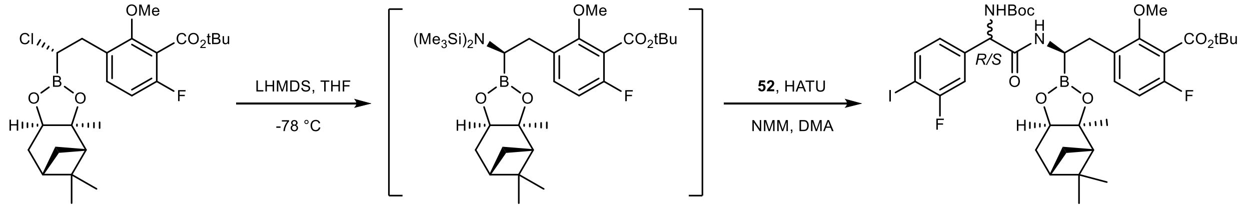

27a
[CAS: 1842401-02-8]
28a
53 (~1:1 mixture)

Scheme S10. Preparation of key amide-containing intermediate **53**.

Step 1: To a solution of **27a** (0.52 g, 1.1 mmol) in THF (4.7 mL) at -78 °C, lithium bistrimethylsilylamide (1 M in THF, 1.2 mL, 1.2 mmol, 1.05 eq) was added. The cold bath was removed, and stirring continued at ambient temperature. After 2 h, the resultant solution of **28a** in THF was used without further purification. Mass spectrum, *m/z* (ESI) 592 $(M)^+$, calculated for $C_{30}H_{51}BFNO_5Si_2$.

Step 2: To a mixture of **28a** (0.88 g, 2.22 mmol, 2 eq) and HATU (0.97 g, 2.56 mmol, 2.3 eq) was added DMA (4.7 mL) followed by NMM (0.31 mL, 2.9 mmol, 2.6 eq). The resulting solution was stirred at ambient temperature. After 90 min, a prepared solution of **52** in THF (*ca.* 6 mL) was added, and the reaction mixture was stirred at ambient temperature. After 18 h, the reaction mixture was diluted with EtOAc, washed with water and brine, dried over anhydrous Na_2SO_4, filtered, and concentrated. The crude product was purified by flash silica gel chromatography (30% EtOAc/hexanes) to obtain **53** (0.64 g, 70%) as an off-white-colored solid. Mass spectrum, *m/z* (ESI) 825 $(M + H)^+$, calculated for $C_{37}H_{49}BF_2IN_2O_8$.

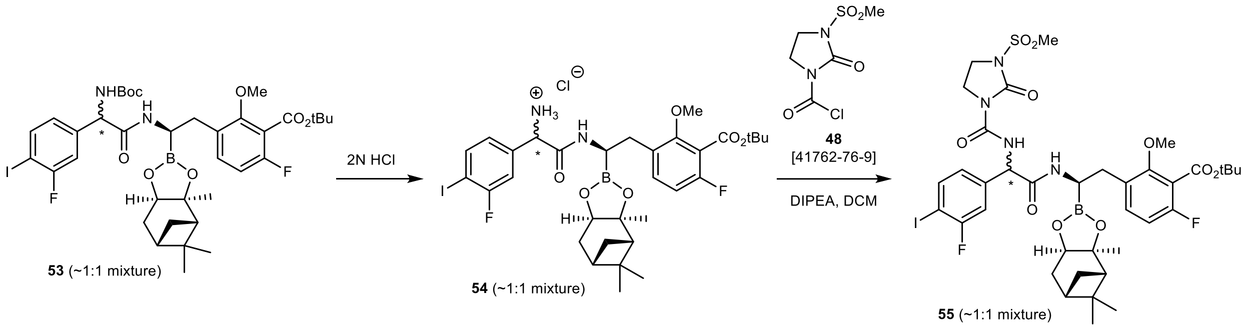

Scheme S11. Preparation of iodophenyl intermediate **55**.

Step 1: To a round-bottomed flask containing **53** (0.64 g, 0.78 mmol) at 0 °C, pre-cooled (0 °C) 2N HCl in diethyl ether (14 mL) was added, and the reaction mixture was slowly warmed to ambient temperature. After 18 h, the reaction mixture was concentrated to provide **54** (0.59 g, quant.) which was used without further purification. Mass spectrum, *m/z* (ESI) 725 $(M + H)^+$, calculated for $C_{32}H_{41}BF_2IN_2O_6$.

Step 2: To a solution of **54** (0.59 g, 0.78 mmol) in CH_2Cl_2 (13 mL) at 0 °C was added DIPEA (0.41 mL, 2.33 mmol, 3 eq) followed by 3-(methylsulfonyl)-2-oxoimidazolidine-1-carbonyl chloride (**48**) and the reaction mixture was stirred at ambient temperature. After 0.5 h, the reaction mixture was washed with water, dried over anhydrous Na_2SO_4, filtered, and concentrated to provide **55** (0.77 g) which was used in the next reaction without further purification. Mass spectrum, m/z (ESI) 915 $(M + H)^+$, calculated for $C_{37}H_{47}BF_2IN_4O_{10}S$.

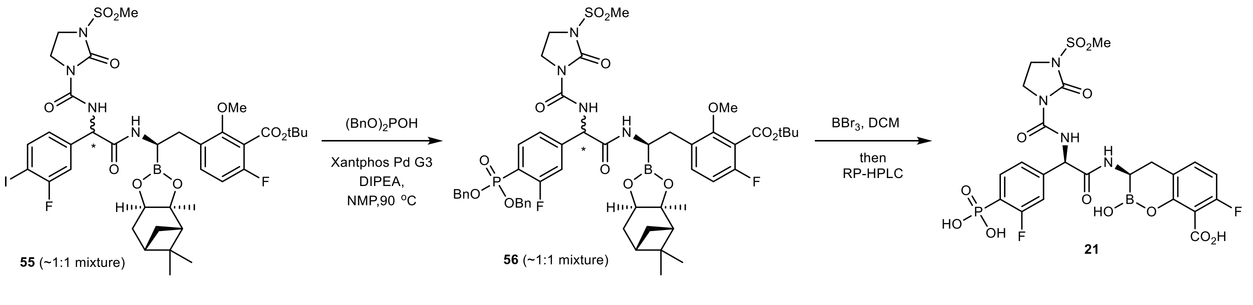

Scheme S12. Preparation of boro-PBPi **21**.

Step 1: To a solution of **55** (0.77 g, 0.84 mmol) in NMP (15 mL), DIPEA (0.44 mL, 2.5 mmol, 3 eq), Xantphos Pd G3 (0.08 g, 0.08 mmol, 10 mol %), and dibenzyl phosphite (0.37 mL, 1.7 mmol,

2 eq) were added, and the reaction mixture was degassed (3×) under argon and then warmed to 90 °C. After 1 h, the reaction mixture was diluted with EtOAc, washed with water and brine, dried over anhydrous Na_2SO_4, filtered, and concentrated to afford **56** (0.74 g) which was used in the next reaction without further purification. Mass spectrum, *m/z* (ESI) 1049 $(M)^+$, calculated for $C_{51}H_{60}BF_2N_4O_{13}PS$.

Step 2: To a solution of **56** (0.74 g, 0.75 mmol) in CH_2Cl_2 (20 mL) at -78 °C, BBr_3 (1M in DCM, 7.5 mL, 7.5 mmol, 10 eq) was added, and the reaction mixture was warmed to ambient temperature. After 18 h, the reaction mixture was cooled to 0 °C, quenched with a solution of water (1.5 mL) and MeOH (0.75 mL), and then concentrated. The crude residue (Peak #2 for *R,R*-diastereomer) was purified by reversed-phase HPLC using a Waters™ XBridge C18 column (5-40% ACN in H_2O containing 0.1% TFA over 15 min; flow rate: 45 mL/min), followed by lyophilization to provide *R,R*-**21** (25 mg) as a white-colored solid. ^1H NMR (400 MHz, methanol-d_4, 27 °C) δ 8.47 (d, *J* = 6.9 Hz, 1H), 7.74 (m, 1H), 7.11 (m, 1H), 7.01 (m, 2H), 6.66 (m, 1H), 5.58 (d, *J* = 6.4 Hz, 1H), 3.86 (m, 2H), 3.77 (m, 2H), 3.30 (m, 4H), 2.87 (s, 2H). Mass spectrum, *m/z* (ESI) 647 $(M + H)^+$, calculated for $C_{22}H_{23}BF_2N_4O_{12}PS$.

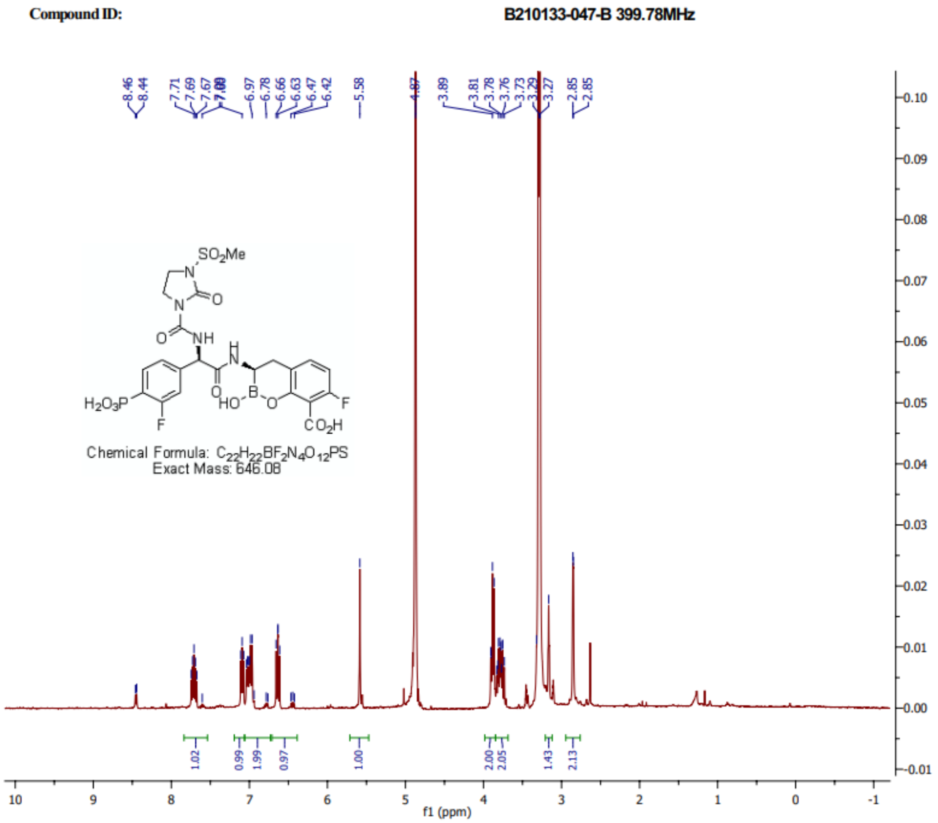

Acquisition Parameters

Date: 2022-03-25T06:46:10
Pulse Sequence:
Temperature: 0 C
Number of Scans: 32
Frequency: 399.78 MHz
Spectral Width: 10016 Hz
Nucleus: Proton
Data Points: 16384
Relaxation Delay: 4 s
Pulse Width: 3.5 us

F2 - Processing Parameters
Data Points: 32768
DC: 0.05
LB: 1.00 Hz
First Point: 0.50
FT: Hyper Invert Quadrature
Phase: Global
Baseline: Bernstein

Figure S12. The ^{1}H NMR spectrum of boro-PBPi **21** in MeOH-d_4.

Print of window 79: MS Spectrum
Data File : C:\USERS\ZHOUX2\DESKTOP\光化学讲座\VNRX-14079-2G\P\B210133-047-AP-37655.D
Sample Name : B210133-047-AP
==
Acq. Operator   : sysadmin
Acq. Instrument : LCMS-A   Location : P1-A-04
Injection Date : 3/9/2022 7:58:55 AM   Inj :   1
                                            Inj Volume : 1.000 µl
Acq. Method   : D:\CHEMSTATION\1\METHODS\P100-1000.M
Last changed   : 4/26/2021 10:26:05 AM by sysadmin
Analysis Method : C:\CHEM32\1\METHODS\DEF_LC.M
Last changed   : 11/20/2006 6:14:44 PM

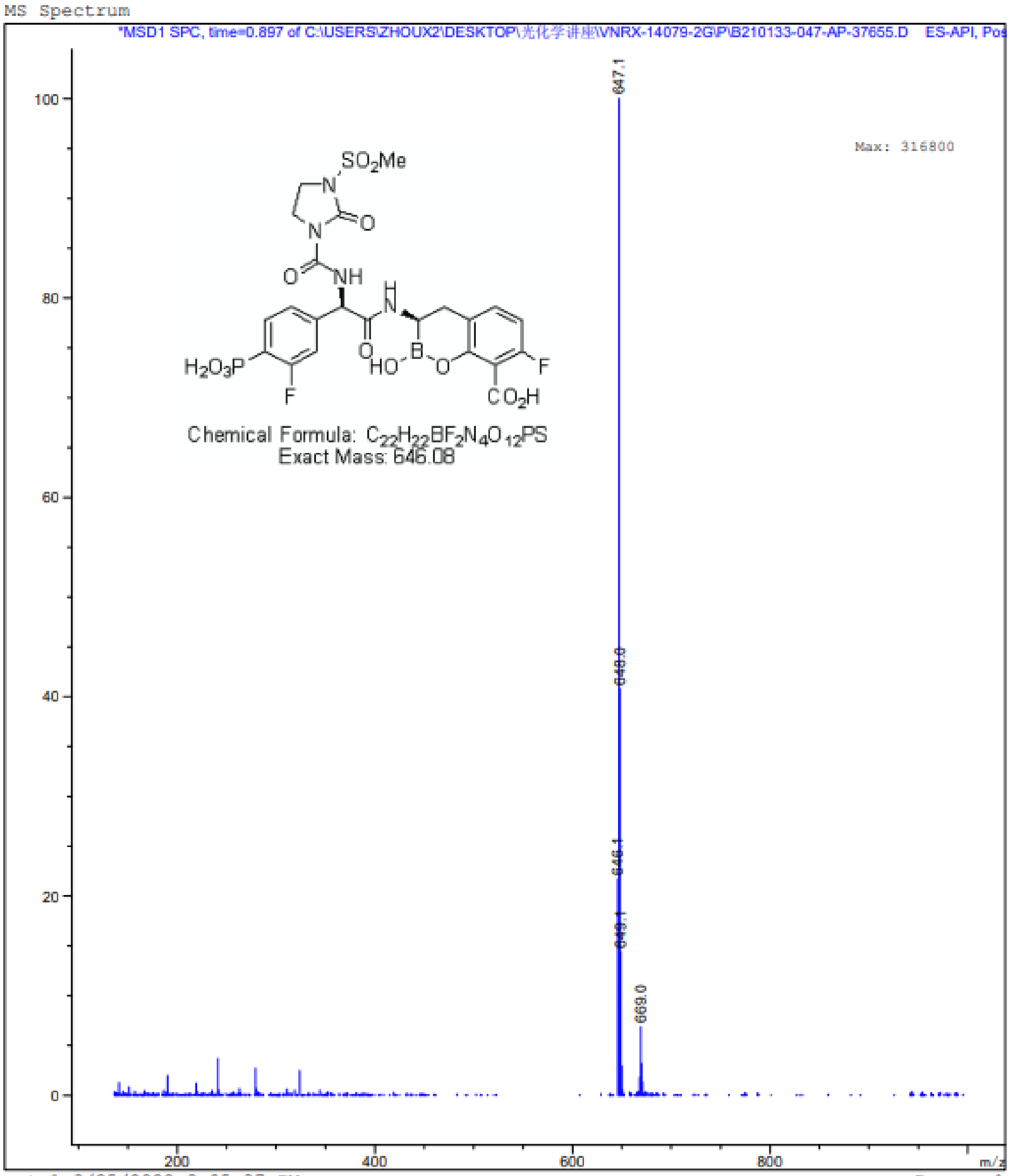

Instrument 1 3/25/2022 3:05:37 PM   Page   1 of 1

Figure S13. Mass spectral data for boro-PBPi **21**.

Table S1. Plasma exposure and pharmacokinetic parameters derived for boro-PBPi **18** following intravenous (IV) and subcutaneous (SC) administration in female BALB/c mice (n = 3/group).

Boro-PBPi 18, plasma exposure (raw data)

**Boro-PBPi 18, IV at 3 mg/kg**						
**Time (h)**	**Concentration (ng/mL)**					
	**Mouse #1**	**Mouse #2**	**Mouse #3**	**Mean**	**SD**	**CV (%)**
0.0833	7350	7560	9100	8003	956	11.9
0.25	3450	4200	4450	4033	520	12.9
0.5	1390	2570	2020	1993	590	29.6
1	489	633	602	575	76	13.2
2	69.3	80.4	74.6	74.8	5.6	7.43
4	15.1	9.07	12.0	12.1	3.0	25.0
8	5.44	8.79	4.72	6.32	2.17	34.4
24	4.08	2.91	BLOQ	2.33	NA	NA

**Boro-PBPi 18, SC at 10 mg/kg**						
**Time (h)**	**Concentration (ng/mL)**					
	**Mouse #4**	**Mouse #5**	**Mouse #6**	**Mean**	**SD**	**CV (%)**
0.0833	12100	8150	9080	9777	2065	21.1
0.25	20000	13700	20000	17900	3637	20.3
0.5	13700	12700	16200	14200	1803	12.7
1	4820	4150	4680	4550	353	7.77
2	340	293	364	332	36	10.9
4	21.4	22.8	36.7	27.0	8.5	31.4
8	19.4	17.1	15.1	17.2	2.2	12.5
24	6.21	2.82	7.30	5.44	2.34	42.9

**Boro-PBPi 18, SC at 30 mg/kg**						
**Time (h)**	**Concentration (ng/mL)**					
	**Mouse #7**	**Mouse #8**	**Mouse #9**	**Mean**	**SD**	**CV (%)**
0.0833	19100	20900	20300	20100	917	4.56
0.25	42800	45400	51800	46667	4632	9.93
0.5	39100	36400	49600	41700	6974	16.7
1	14100	14000	19100	15733	2916	18.5
2	1020	1130	1700	1283	365	28.4
4	85.3	80.7	101	89.0	10.6	12.0
8	29.5	34.3	46.8	36.9	8.9	24.2
24	2.87	2.76	4.85	3.49	1.18	33.7

Boro-PBPi 18, SC at 100 mg/kg						
Time (h)	Concentration (ng/mL)					
	Mouse #10	Mouse #11	Mouse #12	Mean	SD	CV (%)
0.0833	88300	89600	83900	87267	2987	3.42
0.25	157000	124000	126000	135667	18502	13.6
0.5	120000	109000	88600	105867	15933	15.0
1	50800	44400	33000	42733	9016	21.1
2	1370	1180	1940	1497	396	26.4
4	446	409	276	377	89	23.7
8	95.4	129	101	108	18	16.6
24	19.5	11.9	15.3	15.6	3.8	24.5

Boro-PBPi 18, SC at 300 mg/kg						
Time (h)	Concentration (ng/mL)					
	Mouse #13	Mouse #14	Mouse #15	Mean	SD	CV (%)
0.0833	227000	207000	212000	215333	10408	4.83
0.25	383000	413000	371000	389000	21633	5.56
0.5	328000	388000	292000	336000	48497	14.4
1	113000	153000	129000	131667	20133	15.3
2	8640	12400	11700	10913	2000	18.3
4	987	1130	976	1031	86	8.33
8	299	327	259	295	34	11.6
24	24.7	47.5	42.2	38.1	11.9	31.3

Boro-PBPi 18, SC at 1000 mg/kg						
Time (h)	Concentration (ng/mL)					
	Mouse #16	Mouse #17	Mouse #18	Mean	SD	CV (%)
0.0833	445000	465000	418000	442667	23587	5.33
0.25	758000	777000	886000	807000	69072	8.56
0.5	793000	782000	753000	776000	20664	2.66
1	384000	482000	468000	444667	53003	11.9
2	41400	86900	57500	61933	23072	37.3
4	2350	4860	3200	3470	1277	36.8
8	636	821	705	721	93	13.0
24	60.6	144	86.6	97.1	42.7	44.0

BLOQ = below LLOQ (lower limit of quantification), LLOQ = 2 ng/mL

Boro-PBPi 18, pharmacokinetic parameters

3 mg/kg IV

Animal	$t_{1/2}$ (h)	C_0 (ng/mL)	AUC_{Inf} (h*ng/mL)	V_{ss} (L/kg)	CL (mL/min/kg)
Mouse #1	13.4	10726	3287	1.64	15.2
Mouse #2	11.5	10141	3988	0.910	12.5
Mouse #3	1.62	13010	3984	0.324	12.6
**Mean**	**8.82**	**11292**	**3753**	**0.957**	**13.4**
SD	6.31	1516	403	0.658	1.5
CV (%)	71.5	13.4	10.8	68.7	11.5

10 mg/kg SC

Animal	$t_{1/2}$ (h)	T_{max} (h)	C_{max} (ng/mL)	AUC_{inf} (ng*h/mL)	F_{SC} (%)
Mouse #4	10.7	0.250	20000	15346	123
Mouse #5	6.49	0.250	13700	12476	100
Mouse #6	9.81	0.250	20000	15856	127
**Mean**	**9.01**	**0.250**	**17900**	**14559**	**116**
SD	2.24	0.000	3637	1822	15

30 mg/kg SC

Animal	$t_{1/2}$ (h)	T_{max} (h)	C_{max} (ng/mL)	AUC_{inf} (ng*h/mL)	F_{SC} (%)
Mouse #7	4.26	0.250	42800	38664	103
Mouse #8	4.19	0.250	45400	38540	103
Mouse #9	4.65	0.250	51800	49647	132
**Mean**	**4.37**	**0.250**	**46667**	**42284**	**113**
SD	0.25	0.000	4632	6377	17

100 mg/kg SC

Animal	$t_{1/2}$ (h)	T_{max} (h)	C_{max} (ng/mL)	AUC_{inf} (ng*h/mL)	F_{SC} (%)
Mouse #10	4.95	0.250	157000	131491	105
Mouse #11	4.10	0.250	124000	115663	92.5
Mouse #12	5.06	0.250	126000	99697	79.7
**Mean**	**4.70**	**0.250**	**135667**	**115617**	**92.4**
SD	0.522	0.000	18502	15897	12.7

300 mg/kg SC

Animal	$t_{1/2}$ (h)	T_{max} (h)	C_{max} (ng/mL)	AUC_{inf} (ng*h/mL)	F_{SC} (%)
Mouse #13	3.93	0.250	383000	335172	89.3
Mouse #14	4.69	0.250	413000	398135	106
Mouse #15	4.79	0.250	371000	333745	88.9
**Mean**	**4.47**	**0.250**	**389000**	**355684**	**94.8**
SD	0.47	0.000	21633	36771	9.8

1,000 mg/kg SC

Animal	$t_{1/2}$ (h)	T_{max} (h)	C_{max} (ng/mL)	AUC_{inf} (ng*h/mL)	F_{SC} (%)
Mouse #16	4.02	0.500	793000	875275	70.0
Mouse #17	4.42	0.500	782000	1029974	82.3
Mouse #18	4.17	0.250	886000	974336	77.9
**Mean**	**4.20**	**0.417**	**820333**	**959862**	**76.7**
SD	0.21	0.144	57134	78358	6.3

Every $t_{1/2}$ (h) was determined using data from three time points.

Table S2. Plasma exposure and pharmacokinetic parameters derived for boro-PBPi **21** following intravenous (IV) and subcutaneous (SC) administration in male CD-1 mice (n = 3/group).

Boro-PBPi 21, plasma exposure (raw data)

**Boro-PBPi 21_IV at 3 mg/kg**						
**Time (h)**	**Concentration (ng/mL)**					
	**Mouse #13**	**Mouse #14**	**Mouse #15**	**Mean**	**SD**	**CV (%)**
0.0833	6860	7448	6765	7024	370	5.27
0.25	3052	3179	4367	3533	725	20.5
0.5	2025	1465	1810	1767	283	16.0
1	418	301	344	354	59	16.8
2	43.0	51.4	71.4	55.3	14.6	26.4
4	5.44	BLOQ	6.53	3.99	NA	NA
8	BLOQ	BLOQ	BLOQ	NA	NA	NA
24	BLOQ	BLOQ	BLOQ	NA	NA	NA

**Boro-PBPi 21_SC at 10 mg/kg**						
**Time (h)**	**Concentration (ng/mL)**					
	**Mouse #16**	**Mouse #17**	**Mouse #18**	**Mean**	**SD**	**CV (%)**
0.25	7647	8583	8886	8372	646	7.71
0.5	7337	7917	8122	7792	407	5.23
1	4015	4607	4554	4392	328	7.46
2	1043	757	537	779	254	32.6
4	67.5	42.2	31.2	47.0	18.6	39.6
8	4.78	4.02	3.47	4.09	0.65	16.0
24	BLOQ	BLOQ	BLOQ	NA	NA	NA

**Boro-PBPi 21_SC at 30 mg/kg**						
**Time (h)**	**Concentration (ng/mL)**					
	**Mouse #19**	**Mouse #20**	**Mouse #21**	**Mean**	**SD**	**CV (%)**
0.25	24680	30050	24570	26433	3133	11.9
0.5	28860	24580	22120	25187	3411	13.5
1	11740	15600	9746	12362	2976	24.1
2	1881	2790	2236	2302	458	19.9
4	100	214	130	148	59	39.7
8	20.5	20.0	13.8	18.1	3.7	20.7
24	BLOQ	BLOQ	BLOQ	NA	NA	NA

Boro-PBPi 21_SC at 100 mg/kg						
Time (h)	Concentration (ng/mL)					
	Mouse #22	Mouse #23	Mouse #24	Mean	SD	CV (%)
0.25	56450	91700	49960	66037	22461	34.0
0.5	39850	65370	53940	53053	12783	24.1
1	43170	34310	40950	39477	4610	11.7
2	9898	9602	16710	12070	4021	33.3
4	1040	772	1083	965	169	17.5
8	252	195	263	237	37	15.6
24	34.5	31.4	27.9	31.3	3.3	10.6

Boro-PBPi 21_SC at 300 mg/kg						
Time (h)	Concentration (ng/mL)					
	Mouse #25	Mouse #26	Mouse #27	Mean	SD	CV (%)
0.25	172500	199400	168400	180100	16840	9.35
0.5	156000	185700	163000	168233	15526	9.23
1	102000	156200	112700	123633	28707	23.2
2	43070	47650	49850	46857	3459	7.38
4	1805	4542	3557	3301	1386	42.0
8	568	778	581	642	118	18.3
24	94.9	121	86.2	101	18	17.9

BLOQ = below LLOQ (lower limit of quantification), LLOQ = 2 ng/mL

Boro-PBPi 21, plasma pharmacokinetic parameters

3 mg/kg IV

Animal	$t_{1/2}$ (h)*	C_0 (ng/mL)	AUC_{Inf} (h*ng/mL)	V_{ss} (L/kg)	CL (mL/min/kg)
Mouse #1	0.499	10285	3069	0.348	16.3
Mouse #2	0.320	11400	2893	0.306	17.3
Mouse #3	0.532	8420	3161	0.348	15.8
**Mean**	**0.450**	**10035**	**3041**	**0.334**	**16.5**
SD	0.114	1506	136	0.024	0.7
CV (%)	25.3	15.0	4.48	7.28	4.55

10 mg/kg SC

Animal	$t_{1/2}$ (h)*	T_{max} (h)	C_{max} (ng/mL)	AUC_{inf} (ng*h/mL)	F_{SC} (%)
Mouse #16	0.802	0.250	7647	9456	93.3
Mouse #17	0.833	0.250	8583	9845	97.1
Mouse #18	0.868	0.250	8886	9593	94.6
**Mean**	**0.834**	**0.250**	**8372**	**9631**	**95.0**
SD	0.033	0.000	646	197	1.9
CV (%)	3.95	0.00	7.71	2.05	2.05

30 mg/kg SC

Animal	$t_{1/2}$ (h)*	T_{max} (h)	C_{max} (ng/mL)	AUC_{inf} (ng*h/mL)	F_{SC} (%)
Mouse #19	0.987	0.500	28860	28990	95.3
Mouse #20	0.877	0.250	30050	33321	109.6
Mouse #21	0.859	0.250	24570	25536	84.0
**Mean**	**0.908**	**0.333**	**27827**	**29282**	**96.3**
SD	0.069	0.144	2882	3901	12.8
CV (%)	7.65	43.30	10.36	13.32	13.32

100 mg/kg SC

Animal	t1/2 (h)	T_{max} (h)	C_{max} (ng/mL)	AUC_{inf} (ng*h/mL)	FSC (%)
Mouse #22	4.412	0.250	56450	82420	81.3
Mouse #23	4.718	0.250	91700	92300	91.1
Mouse #24	4.060	0.500	53940	94760	93.5
**Mean**	**4.397**	**0.333**	**67363**	**89826**	**88.6**
SD	0.329	0.144	21114	6531	6.4
CV (%)	7.49	43.30	31.34	7.27	7.27

300 mg/kg SC

Animal	$t_{1/2}$ (h)	T_{max} (h)	C_{max} (ng/mL)	AUC_{inf} (ng*h/mL)	F_{SC} (%)
Mouse #25	5.053	0.250	172500	255277	83.9
Mouse #26	4.257	0.250	199400	331224	108.9
Mouse #27	4.152	0.250	168400	280207	92.1
**Mean**	**4.487**	**0.250**	**180100**	**288903**	**95.0**
SD	0.493	0.000	16840	38713	12.7
CV (%)	10.98	0.00	9.35	13.40	13.40

*Calculated half-life. The terminal half-life was not determined because the concentrations at later time points were below the limit of detection.

Table S3. Predicted *f*T>MIC for the doses of boro-PBPi **18** used in in vivo efficacy studies against wild-type *N. gonorrhoeae* strain FA1090 (ATCC 700825), measured using the PK data.

Group	Test article description	Route, schedule	mg/kg each dose	mg/kg/day	End point	Predicted *f*T>MIC (%)
**4**	Boro-PBPi 18	SC, BID	0.25	0.5	26 h	13
**5**	Boro-PBPi 18	SC, BID	7.5	15	26 h	40
**6**	Boro-PBPi 18	SC, BID	50	100	26 h	100
**7**	Boro-PBPi 18	SC, BID	200	400	26 h	100

Table S4. Comparison of groups (p-values) of In vivo efficacy data and comparison of groups for boro-PBPi **21** in the murine vaginal infection model with ceftriaxone-resistant *N. gonorrhoeae* strain H041.

Comparison of groups (p-values)

**Test article**	**PBS**	**Ceftriaxone**	**Boro-PBPi 21**		
	**5 mL/kg SC, q8h**	**120 mg/kg SC, q8h**	**10 mg/kg SC, q12h**	**200 mg/kg SC, q24h**	**150 mg/kg SC, q12h**
**Ceftriaxone 120 mg/kg SC, q8h**	0.6324	-			
**Boro-PBPi 21 10 mg/kg SC, q12h**	**0.0033**	0.9889	-		
**Boro-PBPi 21 200 mg/kg SC, q24h**	**<0.0001**	**0.0059**	0.9376	-	
**Boro-PBPi 21 150 mg/kg SC, q12h**	**<0.0001**	**0.0033**	0.6322	>0.9999	-
**Boro-PBPi 21 150 mg/kg SC, q8h**	**<0.0001**	**0.0008**	0.2277	>0.9999	>0.9999

p-values were determined by two-way ANOVA analysis using log_{10}(CFU/mL) values (significant differences [p <0.05] shown in **bold**). The degrees of freedom and F values calculated from the comparison of groups were F (5, 54) = 13.34.

Table S5. Percentage of polymorphonuclear leukocytes (PMN) in samples from the murine vaginal infection model with ceftriaxone-resistant *N. gonorrhoeae* strain H041.

**PBS (% PMN out of 100 cells)**										
**Days Post-Treatment**	**Mouse #1**	**Mouse #2**	**Mouse #3**	**Mouse #4**	**Mouse #5**	**Mouse #6**	**Mouse #7**	**Mouse #8**	**Mouse #9**	**Mouse #10**
0	6	0	13	3	0	0	0	0	0	4
1	10	0	41	0	0	0	0	0	0	10
2	16	0	55	0	0	0	6	0	0	11
3	21	0	80	0	4	0	4	0	5	21
4	28	29	46	0	5	0	8	61	21	12
5	5	12	48	0	0	0	0	58	48	5
6	3	49	41	0	7	0	0	49	17	30
7	20	11	28	0	17	4	15	50	26	7
8	32	9	50	0	1	0	2	8	18	0

**Ceftriaxone, 120 mg/kg SC, q8h (% PMN out of 100 cells)**										
**Days Post-Treatment**	**Mouse #11**	**Mouse #12**	**Mouse #13**	**Mouse #14**	**Mouse #15**	**Mouse #16**	**Mouse #17**	**Mouse #18**	**Mouse #19**	**Mouse #20**
0	0	0	0	0	0	0	0	0	0	0
1	0	16	4	0	0	0	34	7	0	0
2	0	21	40	0	0	0	39	57	0	25
3	19	58	51	0	7	0	48	68	9	31
4	41	53	45	0	46	3	45	24	14	52
5	3	65	37	0	14	5	29	20	17	54
6	36	52	46	0	34	60	36	23	24	39
7	46	61	67	0	45	32	20	10	26	26
8	53	55	63	7	46	58	21	13	32	40

**Boro-PBPi 21, 10 mg/kg SC, q12h (% PMN out of 100 cells)**										
**Days Post-Treatment**	**Mouse #21**	**Mouse #22**	**Mouse #23**	**Mouse #24**	**Mouse #25**	**Mouse #26**	**Mouse #27**	**Mouse #28**	**Mouse #29**	**Mouse #30**
0	0	3	0	0	0	0	0	0	0	0
1	0	41	0	0	0	13	0	0	0	19
2	0	56	0	33	24	4	0	0	0	44
3	10	76	34	42	17	0	13	0	8	90
4	20	21	15	45	12	0	26	0	8	71
5	24	12	20	34	4	0	34	0	13	56
6	10	70	4	78	13	3	53	0	51	75
7	21	48	38	37	23	5	43	0	6	80
8	49	49	33	32	4	4	45	0	8	81

Boro-PBPi 21, 200 mg/kg SC, q24h (% PMN out of 100 cells)										
Days Post-Treatment	Mouse #31	Mouse #32	Mouse #33	Mouse #34	Mouse #35	Mouse #36	Mouse #37	Mouse #38	Mouse #39	Mouse #40
0	5	0	0	0	0	0	0	0	0	0
1	0	0	0	12	0	26	0	12	3	0
2	0	2	0	33	19	53	6	20	77	1
3	0	3	0	56	50	57	21	41	78	13
4	0	7	0	64	59	37	37	10	57	4
5	0	9	0	30	16	31	43	36	29	4
6	0	0	0	45	18	20	33	22	56	0
7	0	0	0	22	0	34	27	39	65	0
8	0	3	0	44	30	30	0	48	84	0

Boro-PBPi 21, 150 mg/kg SC, q12h (% PMN out of 100 cells)										
Days Post-Treatment	Mouse #41	Mouse #42	Mouse #43	Mouse #44	Mouse #45	Mouse #46	Mouse #47	Mouse #48	Mouse #49	Mouse #50
0	0	0	0	0	4	0	0	0	0	0
1	0	0	0	0	4	25	0	0	0	0
2	0	0	0	0	5	32	4	1	5	10
3	0	0	0	0	0	53	27	67	44	29
4	12	0	0	0	0	57	57	42	31	30
5	0	0	0	0	0	46	18	40	40	30
6	0	0	0	13	0	35	20	51	11	20
7	0	2	0	64	10	32	7	47	34	30
8	0	23	25	65	13	55	3	43	19	33

Boro-PBPi 21, 150 mg/kg SC, q8h (% PMN out of 100 cells)										
Days Post-Treatment	Mouse #51	Mouse #52	Mouse #53	Mouse #54	Mouse #55	Mouse #56	Mouse #57	Mouse #58	Mouse #59	Mouse #60
0	0	0	0	3	0	0	0	2	0	1
1	0	0	0	0	0	5	6	15	0	0
2	53	0	0	0	0	71	21	46	2	13
3	81	0	3	0	0	33	35	52	63	5
4	61	0	0	0	0	46	35	0	29	23
5	59	0	0	0	0	17	16	15	50	0
6	50	3	0	0	0	18	48	50	58	21
7	50	0	0	0	0	38	50	44	23	0
8	19	0	0	3	0	0	50	35	30	0

Table S6. *N. gonorrhoeae* strain description and variants in genes associated with β-lactam resistance.

Strain	Strain information	Source	*penA* allele type (NG-STAR, PBP2)	PBP1	MtrR efflux regulator	mtCDE-mtrR intergenic*	PorB1b porin
**ATCC 49226**	CLSI QC strain	ATCC	*penA-22*	T375A, S666F	A39T	G120bp→A	Not present, PorB1a type porin
**FA19**	Wild type	Robert Nicholas	*penA-15*, reference (WT)	WT	WT	WT	Not present, PorB1a type porin
**FA1090**	ATCC 700825	Ann Jerse	*penA-1*	WT	WT	WT	WT PorB1b
**WHO F**	CDC-0901	CDC	*penA-15*	WT	WT	WT	Not present, PorB1a type porin
**WHO G**	CDC-0902	CDC	*penA-2*	L421P	H105Y	Del_A193bp	Not present, PorB1a type porin
**WHO M**	CDC-0905	CDC	*penA-2*	L421P	G45D	Del_A193bp	23aa variants (key: G120K, A121D)
**MS11**		Ann Jerse	*penA-22*	L421P	A39T	WT	16 aa variants (key: G120D, A121D)
**WHO L**	CDC-0904	CDC	*penA-7*	L421P	G45D	G120bp→A	19 aa variants (key: G120K, A121D)
**WHO K**	CDC-0903	CDC	Mosaic *penA-10*	L421P	G45D	Del_A193bp	22 aa variants (key: G120K, A121D)
**H041**	WHO X, CDC-0912	Ann Jerse	Mosaic *penA-37*	L421P	H105Y	Del_A193bp	28 aa variants (key: G120K, A121D)
**CDC-0914**	WHO Z, A8806	CDC	Mosaic *penA-64*	L421P	A40D, T86A	A193bp→C	21 aa variants (key: G120K, A121D)
**WHO Q**	G7944, NCTC 14208	NCTC	Mosaic *penA-60*	L421P	G45D	Del_A193bp	13 aa variants (key: G120K, A121D)
**CDC-0197**		CDC	Mosaic *penA-34*	L421P	H105Y	Del_A193bp	26 aa variants (key: G120K A121N)
**F89**	WHO Y, CDC-0913	Ann Jerse	Mosaic *penA-42*	L421P	H105Y	Del_A193bp	25 aa variants (key: G120K A121N)

ATCC, American Type Culture Collection; CDC, Centers for Disease Control and Prevention; NCTC, National Collection of Type Cultures; CLSI, Clinical and Laboratory Standard Institute.

Table S7. PBP2 variants in *N. gonorrhoeae* strains.

Strain	*penA* allele type (NG-STAR, PBP2)	Variant No.	A311	I312	V316	D345 ins	T483	A501	G542	G545	P551
**ATCC 49226**	*penA-22*	11	-	-	-	Y	-	-	-	-	-
**FA19**	*penA-15*, reference (WT)	-	-	-	-	-	-	-	-	-	-
**FA1090**	*penA-1*	2	-	-	-	Y	-	-	-	-	-
**WHO F**	*penA-15*	-	-	-	-	-	-	-	-	-	-
**WHO G**	*penA-2*	5	-	-	-	Y	-	-	-	-	-
**WHO M**	*penA-2*	5	-	-	-	Y	-	-	-	-	-
**MS11**	*penA-22*	10	-	-	-	Y	-	-	-	-	-
**WHO L**	*penA-7*	7	-	-	-	Y	-	V	S	-	-
**WHO K**	Mosaic *penA-10*	59	-	M	T	-	-	-	-	S	-
**H041**	Mosaic *penA-37*	61	V	M	P	-	S	-	-	S	V
**CDC-0914**	Mosaic *penA-64*	57	V	M	T	-	S	-	-	S	V
**WHO Q**	Mosaic *penA-60*	49	V	M	T	-	S	-	-	S	V
**CDC-0197**	Mosaic *penA-34*	51	-	M	T	-	-	-	-	S	-
**F89**	Mosaic *penA-42*	52	-	M	T	-	-	P	-	S	-

NG-STAR: the *N. gonorrhoeae* Sequence Typing for Antimicrobial Resistance (NG-STAR) molecular typing scheme[3]

Table S8. Agar MIC (µg/mL) against isogenic mutants related to the PorB porin and the Mtr efflux.

Compound	FA19				FA19 penA4			FA6140 (*penA-4 penB ponA1 mrtR171)*	
	Parent	*mtrR171 (efflux↑)*	*porB*$_{FA1090}$	*porB*$_{FA1090}$-G120K	*porB*$_{FA1090}$	*mtrR171 porB*$_{FA1090}$	*mtrR171 porB*$_{FA1090}$-ΔG120ΔA121	Parent	*mtrD::kan* (Δefflux)
**Azithromycin**	0.06	0.25	0.06	1	0.06	0.25	0.25	0.12	0.016
**Zoliflodacin**	0.03	0.25	0.03	0.03	0.03	0.03	0.12	0.12	≤0.004
**Ceftriaxone**	≤0.002	≤0.002	≤0.002	≤0.002	0.004	0.008	0.004	0.12	0.008
**Boro-PBPi 1**	4	4	4	4	4	8	8	32	16
**Boro-PBPi 12**	≤0.016	0.03	≤0.016	≤0.016	0.06	0.5	0.5	2	0.12
**Boro-PBPi 15**	0.008	≤0.004	≤0.004	0.008	0.016	0.03	0.03	0.25	0.03
**Boro-PBPi 18**	0.008	0.008	0.008	0.008	0.016	0.06	0.06	0.25	0.03
**Boro-PBPi 21**	0.008	0.008	0.008	0.008	0.016	0.03	0.016	0.12	0.06

Table S9. Boro-PBPi activity against zoliflodacin-resistant mutants generated in strain WHO K.

Strain	15	18	21	CRO	ZOLI	GEPO	CIP	AZM	WGS result
**WHO K Parent**	0.5	0.5	0.25	0.03	0.12	0.5	64	0.25	-
**WHO K Colony 3**	0.5	0.25	0.12	0.03	4	0.25	32	0.06	GyrB D429N
**WHO K Colony 7**	0.25	0.25	0.12	0.03	4	0.25	32	0.06	GyrB D429N
**WHO K Colony 10**	0.5	0.25	0.12	0.06	4	0.25	64	0.06	GyrB D429N
**WHO K Colony 13**	0.5	0.25	0.12	0.03	4	0.25	32	0.12	GyrB D429N

Broth MIC (µg/mL)

Abbreviations: CRO, ceftriaxone; ZOLI, zoliflodacin; GEPO, gepotidacin; AZM, azithromycin; CIP, ciprofloxacin; PEN G, penicillin G; WGS, whole genome sequence.

Table S10. Agar MIC (µg/mL) against each of the 44 CDC *N. gonorrhoeae* strains.

Strain ID	*penA* allele	12	15	18	21	CRO	ZOLI	GEPO	AZM	CIP	PEN G
**CDC-0166**	*penA*-34 mosaic	2	0.5	0.5	0.12	0.06	0.12	0.5	0.5	16	2
**CDC-0167**	*penA*-2	0.06	0.016	0.016	0.016	0.004	0.06	0.25	8	≤0.004	0.12
**CDC-0168**	*penA*-34 mosaic	2	0.12	0.12	0.06	0.06	0.06	0.25	0.25	16	1
**CDC-0169**	*penA*-34 mosaic	4	0.25	0.25	0.12	0.06	0.12	0.5	0.5	16	1
**CDC-0170**	*penA*-34 mosaic	2	0.25	0.25	0.06	0.06	0.12	0.5	0.25	16	1
**CDC-0171**	*penA*-34 mosaic	2	0.12	0.12	0.06	0.06	0.06	0.5	0.25	16	2
**CDC-0172**	*penA*-34 mosaic	2	0.25	0.5	0.12	0.06	0.06	0.5	0.25	16	1
**CDC-0173**	*penA*-34 mosaic	4	0.25	0.5	0.12	0.06	0.12	0.5	0.5	16	1
**CDC-0174**	*penA*-34 mosaic	4	0.5	0.5	0.12	0.12	0.12	0.5	0.5	16	2
**CDC-0175**	*penA*-2	0.06	0.016	0.016	0.016	0.004	0.12	0.5	4	≤0.004	0.12
**CDC-0176**	*penA*-34 mosaic	2	0.25	0.25	0.06	0.06	0.12	1	0.25	16	2
**CDC-0177**	*penA*-2	0.25	0.03	0.03	0.03	0.008	0.25	1	1	0.016	0.5
**CDC-0178**	*penA*-34 mosaic	4	0.5	0.5	0.06	0.06	0.12	0.25	0.5	16	2
**CDC-0179**	*penA*-2	0.12	0.016	0.016	0.016	0.004	0.06	0.25	8	≤0.004	0.12
**CDC-0180**	*penA*-34 mosaic	4	0.5	0.5	0.12	0.12	0.12	0.25	0.25	16	2
**CDC-0182**	*penA*-34 mosaic	4	0.5	0.5	0.12	0.12	0.12	1	0.5	16	2
**CDC-0183**	*penA*-34 mosaic	4	0.25	0.25	0.06	0.06	0.12	0.5	1	32	4
**CDC-0184**	*penA*-34 mosaic	2	0.25	0.25	0.06	0.06	0.12	1	0.25	16	2
**CDC-0185**	*penA*-34 mosaic	2	0.25	0.5	0.06	0.12	0.12	1	0.25	16	2
**CDC-0186**	*penA*-34 mosaic	4	0.5	0.5	0.12	0.12	0.06	0.25	0.25	16	2
**CDC-0187**	*penA*-34 mosaic	1	0.12	0.12	0.06	0.03	0.25	1	1	8	0.5
**CDC-0188**	*penA*-34 mosaic	2	0.25	0.25	0.06	0.06	0.12	0.5	0.25	16	2
**CDC-0189**	*penA*-34 mosaic	2	0.25	0.25	0.06	0.06	0.12	1	0.25	16	2
**CDC-0190**	*penA*-34 mosaic	4	0.5	0.5	0.12	0.12	0.06	1	0.25	16	1
**CDC-0191**	*penA*-34 mosaic	2	0.5	0.5	0.06	0.12	0.12	1	0.5	16	4
**CDC-0192**	*penA*-34 mosaic	2	0.25	0.5	0.06	0.06	0.12	0.5	0.5	16	2
**CDC-0193**	*penA*-9 (*penA*-2 + P551L)	2	0.5	0.5	0.12	0.12	0.25	1	1	0.016	2
**CDC-0195**	*penA*-34 mosaic	4	0.25	0.25	0.12	0.06	0.12	1	0.5	16	2
**CDC-0196**	*penA*-34 mosaic	2	0.5	0.5	0.12	0.12	0.12	0.5	0.5	16	2
**CDC-0197**	*penA*-34 mosaic	2	0.25	0.25	0.06	0.03	0.06	0.25	4.1	16	1
**CDC-0198**	*penA*-34 mosaic	2	0.25	0.25	0.06	0.06	0.12	0.5	0.5	2	2
**CDC-0199**	*penA*-9 (*penA*-2 + P551L)	1	0.25	0.25	0.12	0.06	0.25	0.5	0.5	1	1
**CDC-0200**	*penA*-34 mosaic	8	0.5	0.5	0.25	0.12	0.12	0.5	0.5	8	2
**CDC-0201**	*penA*-34 mosaic	2	0.25	0.25	0.06	0.06	0.12	0.5	0.25	2	2
**CDC-0203**	*penA*-34 mosaic	4	0.5	0.5	0.12	0.06	0.12	0.5	0.25	4	2
**CDC-0204**	*penA*-34 mosaic	2	0.5	0.25	0.06	0.25	0.12	1	0.25	2	2
**CDC-0206**	*penA*-34 mosaic	2	0.5	0.5	0.06	0.06	0.12	1	0.25	2	1
**CDC-0207**	*penA*-34 mosaic	2	0.5	0.5	0.12	0.12	0.12	0.5	0.5	2	1
**CDC-0208**	*penA*-34 mosaic	2	0.25	0.25	0.06	0.06	0.12	0.5	0.5	2	2
**CDC-0209**	*penA*-34 mosaic	2	0.25	0.25	0.06	0.06	0.12	1	0.5	2	2
**CDC-0210**	*penA*-34 mosaic	4	0.25	0.5	0.12	0.06	0.12	1	0.25	4	1
**CDC-0212**	*penA*-34 mosaic	4	0.5	0.5	0.12	0.06	0.12	1	0.5	16	2
**CDC-0213**	*penA*-34 mosaic	4	0.25	0.5	0.06	0.06	0.12	0.5	0.12	16	1
**CDC-0214**	*penA*-34 mosaic	4	0.25	0.25	0.12	0.12	0.12	0.5	0.25	16	2
**Summary**		**12**	**15**	**18**	**21**	**CRO**	**ZOLI**	**GEPO**	**AZM**	**CIP**	**PEN G**
**MIC_{50}**		2	0.25	0.25	0.06	0.06	0.12	0.5	0.5	16	2
**MIC_{90}**		4	0.5	0.5	0.12	0.12	0.12	1	1	16	2
**MIC_{100}**		8	0.5	0.5	0.25	0.25	0.25	1	8	32	4

This strain panel consists of isolates from surveillance studies in the United States with diverse antimicrobial susceptibilities to a variety of known drugs used to treat gonorrhea[4]. The analysis of the genomes published previously[4] showed that most strains carried *penA-34* and *penA-2*. Abbreviations: CRO, ceftriaxone; ZOLI, zoliflodacin; GEPO, gepotidacin; AZM, azithromycin; CIP, ciprofloxacin; PEN G, penicillin G.

Table S11. Frequency of Resistance (FoR) data of boro-PBPi **21** and ceftriaxone.

Compound	Boro-PBPi 21		Ceftriaxone	
**Concentration**	**4× MIC**	**16× MIC**	**4× MIC**	**16× MIC**
**ATCC 49226**	$<1.56 \times 10^{-9}$	$<1.56 \times 10^{-9}$	$<1.56 \times 10^{-9}$	$<1.56 \times 10^{-9}$
**H041**	$<1.79 \times 10^{-9}$	$<1.79 \times 10^{-9}$	$<1.79 \times 10^{-9}$	$<1.79 \times 10^{-9}$
**WHO Q**	$<4.17 \times 10^{-9}$	$<4.17 \times 10^{-9}$	$<4.17 \times 10^{-9}$	$<4.17 \times 10^{-9}$
**WHO L**	$<1.09 \times 10^{-9}$	$<1.09 \times 10^{-9}$	$<1.09 \times 10^{-9}$	$<1.09 \times 10^{-9}$
**CDC-0197**	$<1.67 \times 10^{-9}$	$<1.67 \times 10^{-9}$	$<1.67 \times 10^{-9}$	$<1.67 \times 10^{-9}$

Table S12. Antibiotic susceptibility and whole genome sequence analysis for mutants isolated from CDC-0197 and WHO L

Strain	Description	**Boro-PBPi 21** MIC (μg/mL)	**CRO** MIC (μg/mL)	**Zoliflodacin** MIC (μg/mL)	**Effect caused by mutation**
**CDC-0197**	**Parent**	0.06	0.03	0.06	Not applicable
	**Day 8 mutant**	8	0.12	0.06	PBP2(Y543C)
**WHO L**	**Parent**	0.25	0.5	0.12	Not applicable
	**Day 8 mutant**	>16	0.12	≤0.016	Truncations of MltE, opacity family porins, AAA family ATPase

The mutant strains of CDC-0194 and WHO L were isolated at day 8 from the 10-day passaging of the parent strains at sub-MIIC as shown in **Extended Data Figure 2**. The genome sequencing analysis was performed as described in the Method.

MICs of **21**, CRO, and zoliflodacin against the parent and mutant strains are presented with the outcome of the genome sequencing analysis. The CDC-0194 mutant had an additional mutation that causes the substitution of Tyr543 with Cys in the mosaic PBP2. In the WHO L mutant, mutations causing the deletion of at least eight genes were identified, whereas no mutation was found in the PBP2 gene (*penA*). One of the mutations causes a truncation of MtrE (NCBI locus tag: C7S00_RS02740 on the NCBI reference sequence ID: GCF_900087875.2), the outer membrane component of the MtrCDE efflux pump, and is likely to be the factor that decreased the zoliflodacin MIC. In addition, the analysis identified frameshift mutations in the genes encoding opacity family porins (C7S00_RS06610, C7S00_RS07350, C7S00_RS00430, C7S00_RS03255, C7S00_RS12125, and C7S00_RS08205) and AAA family ATPase (C7S00_RS03675).

Table S13. Safety and selectivity data for boro-PBPi **21**.

a. Plasma protein binding (mouse and human)

Mouse plasma protein binding data:

Compound	Free (%)	Bound (%)		Stability (%)		Recovery (%)	
	Replicate	Replicate	Mean	Replicate	Mean	Replicate	Mean
**Boro-PBPi 21**	82.68	17.32	18.97	94.58	95.47	93.11	93.13
	79.38	20.62		96.36		93.15	
**Warfarin**	3.23	96.77	96.62	96.21	98.11	89.90	89.02
	3.52	96.48		100.00		88.15	

Human plasma protein binding data:

Compound	Free (%)	Bound (%)		Stability (%)		Recovery (%)	
	Replicate	Replicate	Mean	Replicate	Mean	Replicate	Mean
**Boro-PBPi 21**	59.98	40.02	39.46	89.63	88.56	83.69	86.45
	61.09	38.91		87.49		89.21	
**Warfarin**	1.14	98.86	98.79	99.65	97.53	89.13	86.98
	1.29	98.71		95.41		84.82	

The plasma protein binding assays were performed by BioDuro-Sundia using the equilibrium dialysis method by incubating the compound at 5 μM with plasma (mouse or human) in one compartment and phosphate buffer in the other compartment separated by a dialysis membrane (12–14 K MWCO) for 5 h at 37 °C with shaking. The amount of compound in each chamber compartment (buffer and plasma) was measured using reverse-phase high-performance liquid chromatography/tandem mass spectrometry methods that were customized for each compound. The data were generated by BioDuro-Sundia.

b. Cytotoxicity

Compound	Concentration tested (μg/mL)	MRC-5 MTS readout (OD_{490} value)	HeLa MTS readout (OD_{490} value)	3T3 MTS readout (OD_{490} value)
**Boro-PBPi 21**	256	0.439	0.505	0.365
	81	0.588	0.455	0.348
	25.6	0.621	0.442	0.354
	8.1	0.511	0.396	0.338
**Chlorohexidine (positive control)**	256	0.115	0.161	0.191
	81	0.103	0.205	0.103
	25.6	0.083	0.146	0.256
	8.1	0.528	0.077	0.480
**DMSO control (average ± standard deviation)**	-	0.457 ± 0.094	0.411 ± 0.077	0.381 ± 0.021
**Medium only (no cells) (average ± standard deviation)**	-	0.050 ± 0.001	0.051 ± 0.002	0.056 ± 0.005

The half maximal cytotoxic concentration (CC_{50}) of each compound against three human cell lines (MRC-5 [ATCC CCL-171], HeLa [ATCC CCL-2], and 3T3-Swiss albino [ATCC CCL-92]) was measured using an MTS-based cell proliferation assay, in which actively dividing cells were used to maximize assay sensitivity. Procedurally, 180 μL of cells were seeded in cell-culture-treated 96-well plates (Celltreat, Pepperell, MA, USA) at a density of 2–3.5 × 10^4 cells/mL and incubated overnight at 37 °C in a humidified 5% CO_2 atmosphere. The next day, the compound was diluted in PBS with 6% DMSO in a 0.5 $\log_{10}$ dilution scheme at 10× the final compound concentration array. Compound titrations (in 20 μL volume) were added to each well of the cell culture plates to achieve a final 10-fold dilution ranging from 8.1–256 μg/mL for boro-PBPi **21**. The final DMSO concentration in all the assays was 0.6%. After an additional 72 h of incubation (when cells reached 90–100% confluency), CellTiter 96® AQ$_{ueous}$ solution (Promega, Fitchburg, WI, USA) was added at 20 μL per well, and plates were incubated for an additional 2–4 h at 37 °C, 5% CO_2. The optical density of the wells was measured using a Biotek Cytation 3 plate reader at 490 nm. The background signal (wells receiving Cell Titer reagent but containing no cells) was subtracted from all wells on the plate. If the cell absorbance was ≥50% compared to the DMSO control wells, the

compound was considered to have no cytotoxicity, and the CC_{50} was recorded as greater than the highest concentration tested. If the cell absorbance was <50% compared to the cell control wells, the percent growth versus $\log_{10}$ drug concentration was plotted, and the concentrations at which 50% of the cells survived compared to the growth control (CC_{50}) were calculated using a sigmoidal four-parameter curve fit in GraphPad Prism.

c. Hemolysis

Compound	Concentration tested	OD541 value	OD541 background (DMSO/water only)	% Hemolysis
**Boro-PBPi 21**	1 mg/mL	0.058	0.079	-0.99
**Water**	-	2.203	0.000	100.00

Human red blood cells (Rockland Immunochemicals, Inc., #R4070050) were diluted in PBS to obtain a target blood cell concentration of 5×10^8 erythrocytes/mL. Desired test compound stock solutions were prepared in 1.2 mL PBS, along with appropriate controls. Diluted blood cells (300 μL) were added to each tube and gently inverted to mix. The tubes were incubated for 1 h at 37 °C, gently inverted to mix, and centrifuged at 11,000 × *g* for 5 min. The optical density (OD) at 541 nm of the supernatant was measured for each desired test sample and control, and the % hemolysis was calculated. In vitro hemolysis values of <10% are considered nonhemolytic[5].

d. Mitochondrial toxicity

Compound	Media	IC_{50} (μM)	Fold shift (Glu/Gal)
**Antimycin**	Glucose	>25	>3
	Galactose	0.00205	
**Staurosporine**	Glucose	0.80270	0.70
	Galactose	1.15300	
**Boro-PBPi 21**	Glucose	>100	Not applicable
	Galactose	>100	

The assay for monitoring mitochondrial toxicity was performed by BioDuro-Sundia. Briefly, SKOV-3 cells (ATCC HTB-77) were seeded and cultured in media containing either glucose or galactose overnight. Test compounds [Boro-PBPi **21**, antimycin, and staurosporine] were added at six concentrations (threefold serial dilution from 100 μM to 0.4 μM) and incubated with the cells for 24 h. Cell viability was measured using the CellTiter-Glo® method (Promega, Fitchburg, WI, USA).

e. Chromosomal aberrations (micronucleus assay)

Micronucleus in CHO-K1 cells without S9

Compound	Concentration (μM)	Scored Cells (Number of Binucleated Cells)		% Micronucleated Cells		% Cytotoxicity CBPI Index		t-Test p-value	Test Result
Boro-PBPi 21	300	13216	13916	1.97%	2.34%	3.77	4.46	0.4730	-
	100.000	14274	14221	2.23%	2.01%	2.38	3.36	0.5003	-
	33.333	13899	14177	2.26%	2.14%	0.52	2.63	0.3105	-
	11.111	13607	14643	2.49%	2.19%	0.37	1.78	0.1847	-
	3.704	13839	14309	2.23%	1.86%	1.94	1.99	0.7625	-
	1.235	14225	14642	1.98%	1.96%	-0.72	2.32	0.9905	-
	0.412	14176	14076	2.19%	2.14%	0.78	2.19	0.3625	-
	0.137	14301	14562	2.17%	1.94%	-0.64	1.15	0.6981	-

Compound	Concentration (μM)	Scored Cells	% Micronucleated Cells	% Cytotoxicity CBPI Index	Test Result
Untreated control	-	14574	2.15%	3.24	NA
	-	14693	2.06%	-2.17	
	-	14998	1.69%	-1.08	
Mitomycin C	1.85	4277	13.82%	62.34	+
	1.85	4744	13.76%	61.80	
	1.85	5006	13.82%	58.95	

Micronucleus in CHO-K1 cells with S9

Compound	Concentration (μM)	Scored Cells (Number of Binucleated Cells)		% Micronucleated Cells		% Cytotoxicity CBPI Index		t-Test p-value	Test Result
Boro-PBPi 21	300	4759	5332	3.99%	3.68%	4.33	6.52	0.3734	-
	100.000	4429	5334	5.08%	4.48%	12.30	4.88	0.0244	-
	33.333	4437	3433	4.28%	6.00%	12.98	18.25	0.1056	-

Compound	Concentration (μM)	Scored Cells (Number of Binucleated Cells)		% Micronucleated Cells		% Cytotoxicity CBPI Index		t-Test p-value	Test Result
	11.111	4650	3116	3.78%	5.55%	12.81	34.47	0.2223	-
	3.704	3577	3584	4.75%	5.69%	16.77	29.55	0.0254	-
	1.235	3222	3953	5.12%	5.89%	24.93	26.16	0.0106	-
	0.412	4351	3370	3.40%	5.93%	13.50	24.78	0.3570	-
	0.137	3913	3684	4.75%	6.30%	20.22	28.65	0.0501	-

Compound	Concentration (μM)	Scored Cells	% Micronucleated Cells	% Cytotoxicity CBPI Index	Test Result
**Untreated control + S9**	-	5453	3.70%	5.91	NA
	-	5604	3.39%	-3.43	
	-	5538	3.79%	-2.48	
**Mitomycin C**	22.375	1669	13.48%	56.08	+
	22.375	1868	10.76%	60.22	
	22.375	1709	10.94%	48.35	

The micronucleus assay to monitor the genotoxicity of boro-PBPi **21** was performed by BioDuro-Sundia as described previously[6]. Briefly, CHO-K1 cells (ATCC CCL-61)) were treated with **21** at concentrations up to 300 μM, with and without incubation with liver S9 fraction, followed by incubation for 24 h. The cells were fixed with 4% paraformaldehyde and stained with 4,6-diamidino-2-phenylindole (DAPI). The micronucleus rate in CHO cells was evaluated using a high-throughput screening system. As positive controls, CHO-K1 cells were incubated with cyclophosphamide with S9 and mitomycin C without S9, and chromosomal damage and micronucleated cells were observed relative to untreated controls.

f. CYP450 inhibition data

CYP450 enzyme	Substrate used	% Enzyme Activity (Relative to DMSO controls) Boro-PBPi 21 at 30 μM		Positive control compound used	Positive control IC_{50} (μM)
		Data 1	Data 2		
**CYP1A2**	Vivid™ EOMCC	96.16	97.67	**Furafylline**	1.70
**CYP2C19**	Vivid™ EOMCC	64.27	66.47	**Ketoconazole**	2.96

CYP450 enzyme	Substrate used	% Enzyme Activity (Relative to DMSO controls)		Positive control compound used	Positive control IC_{50} (μM)
		Boro-PBPi 21 at 30 μM			
		Data 1	Data 2		
**CYP2D6**	Vivid™ EOMCC	98.28	103.14	**Ketoconazole**	9.59
**CYP3A4**	Vivid™ BOMCC	100.61	102.47	**Ketoconazole**	0.0052
**CYP2E1**	Vivid™ EOMCC	92.83	95.60	**Tranylcypromine**	91.9
**CYP2B6**	Vivid™ BOMCC	107.41	109.00	**Miconazole**	0.036
**CYP2C9**	Vivid™ OOMR	96.60	95.24	**Ketoconazole**	3.03
**CYP2C8**	Vivid™ DBOMF	101.96	103.27	**Miconazole**	3.76

The inhibition of CYP450 enzymes was monitored using boro-PBPi **21** at 30 μM in fluorescence-based assays using Vivid™ substrates relative to DMSO controls. The IC_{50}s of the positive control compounds were also determined to validate the assays. The assays were performed at Reaction Biology (Malvern, PA, USA).

g. hERG binding data

Compound	% tracer binding		Positive control IC_{50} (μM)
	Data 1	Data 2	
**DMSO**	102.20	95.48	ND
**Boro-PBPi 21 at 30 μM**	88.32	94.99	ND
**E4031 (positive control)**	NA	NA	0.019

The binding of boro-PBPi **21** to hERG in the membrane was monitored using a fluorescence-based assay with 1 nM Predictor™ hERG Tracer Red and 1X Predictor™ hERG membrane. Fluorescence was measured using excitation and emission wavelengths of 531 and 595 nm, respectively. The assay was performed at Reaction Biology (Malvern, PA, USA).

h. Protease inhibition data

Target:	% Enzyme Activity (relative to DMSO controls)		Control compound	Control Compound IC_{50} (M)
	Boro-PBPi 21 at 30 µM			
	Data 1	Data 2		
**Chymotrypsin**	82.12	82.35	**Chymostatin**	7.27E-10
**Thrombin a**	89.45	91.98	**Gabexate mesylate**	1.49E-06
**Trypsin**	94.04	90.85	**Gabexate mesylate**	2.40E-08

Protease activity was monitored as a time-course measurement of the increase in fluorescence signal from the fluorescently labeled peptide substrate, and the initial linear portion of the slope (signal/min) was analyzed. The assays were performed at Reaction Biology (Malvern, PA, USA).

i. Thermodynamic solubility

Compound	Solubility (µM) in buffer (pH 7.4)	Target concentration (µM)
**21**	732.0	773.7

The thermodynamic solubility of boro-PBPi **21** was determined by BioDuro-Sundia. **21** (1.18 mg) was mixed with 2.36 mL of 50 mM potassium phosphate buffer (pH 7.4) and incubated for 16 h at room temperature with shaking at 1000 rpm, followed by static incubation for 30 min at room temperature. The supernatant was collected, and the amount of **21** in the supernatant was measured using reverse-phase high-performance liquid chromatography/tandem mass spectrometry.

j. Plasma stability (human and mouse)

Compound	$T_{1/2}$ (min)	
	Mouse	Human
**21**	>372.8	>372.8
**Propantheline**	41.6	25.6

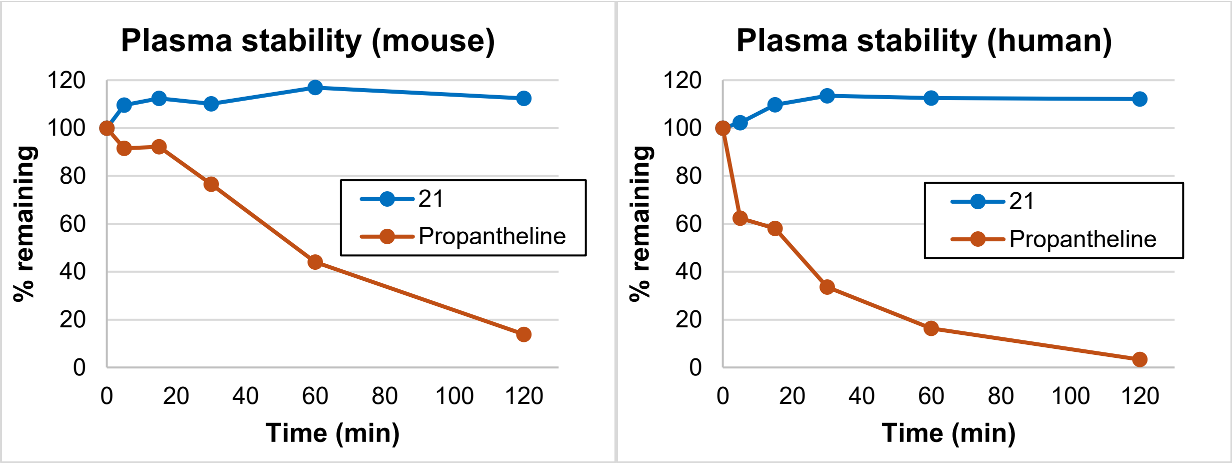

The plasma stability assay was performed by BioDuro-Sundia. The compound (**21** or control compound: propantheline) at 1 μM was incubated with plasma (mouse or human) at 37 °C with shaking at 100 rpm. Samples were taken at different time points (0, 5, 15, 30, 60, and 120 min for the control compound and 0, 15, 30, 60, and 120 min for **21**), mixed with quenching solution (5/5/10 ng/mL of buspirone/terfenadine/tolbutamide in MeOH/acetonitrile (1:3, v/v)), and centrifuged at 4,000 rpm for 15 min at 4 °C. The compound amounts in the supernatants were measured using LC-MS/MS.

k. Hepatocytes metabolism stability (mouse and human)

Compound	Mouse			
	$T_{1/2}$ (min)	In vitro CL_{int} (μL/min/million cells)	CL_{hep} (mL/min/kg)	Extraction ratio
**21**	>372.8	<1.9	<16.0	<0.18
**Diclofenac**	29.2	23.7	66.1	0.73
**7-EC**	<4.5	>153.5	>85.2	>0.95

Compound	Human			
	$T_{1/2}$ (min)	In vitro CL_{int} (μL/min/million cells)	CL_{hep} (mL/min/kg)	Extraction ratio
**21**	>372.8	<1.9	<4.5	<0.22
**7-EC**	6.06	114	19.6	0.94

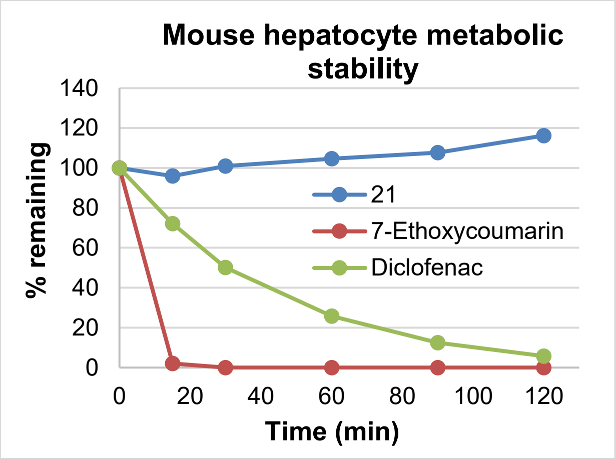

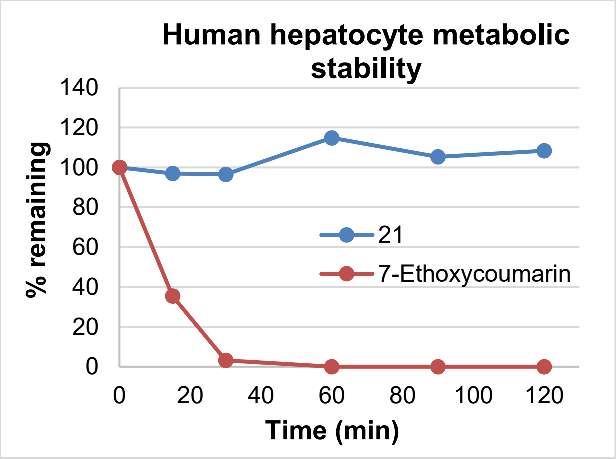

Hepatocyte metabolism stability assay was performed by BioDuro-Sundia. The compound (**21** or control compounds: 7-ethoxycoumarine and diclofenac) at 1 μM was incubated with mouse or human hepatocytes (1 × 10^6 cells/mL) in the incubation medium (Williams E medium supplemented with 4 mM glutamine) at 37 °C with shaking at 100 rpm. Samples were collected at different time points (0, 15, 30, 60, 90, and 120 min), mixed with a quenching solution (5/5/10 ng/mL of buspirone/terfenadine/tolbutamide in MeOH/acetonitrile (1:3, v/v)), and centrifuged at 4,000 rpm for 15 min at 4 °C. The compound amounts in the supernatants were measured using LC-MS/MS methods. The values of in vitro CL_{int}, CL_{hep}, and extraction ratio were calculated as described previously[7].

Table S14. Bacterial strains used.

Species	Strain ID	Strain information	Source/reference
N. gonorrhoeae	ATCC 49226	CLSI QC strain	ATCC
N. gonorrhoeae	FA19	Wild type, penicillin-susceptible laboratory strain	Robert Nicholas[8]
N. gonorrhoeae	FA1090	ATCC 700825	Ann Jerse[9]
N. gonorrhoeae	WHO F	CDC-0901	CDC[10]
N. gonorrhoeae	WHO G	CDC-0902	CDC[10]
N. gonorrhoeae	WHO M	CDC-0905	CDC[10]
N. gonorrhoeae	MS11		Ann Jerse[11]
N. gonorrhoeae	WHO L	CDC-0904	CDC[10]
N. gonorrhoeae	WHO K	CDC-0903	CDC[10]
N. gonorrhoeae	H041	WHO X, CDC-0912	Ann Jerse
N. gonorrhoeae	CDC-0914	WHO Z, A8806	CDC
N. gonorrhoeae	WHO Q	G7944, NCTC 14208	NCTC[12]
N. gonorrhoeae	CDC-0197	12AZI_T_003	CDC
N. gonorrhoeae	F89	WHO Y, CDC-0913	Ann Jerse[13]
N. gonorrhoeae	H041(STR^R)	H041 streptomycin-resistance, used in the in vivo efficacy study	Ann Jerse[14]
N. gonorrhoeae	FA19 *porB*$_{FA1090}$(wt)		Robert Nicholas[15]
N. gonorrhoeae	FA19 *porB*$_{FA1090}$(G120K)		Robert Nicholas[15]
N. gonorrhoeae	FA19 *penA4 mtrR171 porB*$_{FA1090}$(wt)		Robert Nicholas[15]
N. gonorrhoeae	FA19 *penA4 mtrR171 porB*$_{FA1090}$(G120K)		Robert Nicholas[15]
N. gonorrhoeae	FA19 *penA4 mtrR171 porB*$_{FA1090}$(ΔG120ΔA121)		Robert Nicholas[15]
N. gonorrhoeae	WV30	FA19 *mtrR171*	Robert Nicholas[16]
N. gonorrhoeae	FA6140	*mtrR171 penA4 penB ponA1*	Robert Nicholas[17]
N. gonorrhoeae	WV22	FA6140 *mtrD*::kan	Robert Nicholas[16]
N. gonorrhoeae	CDC set	44 CDC strains	CDC[4]
N. gonorrhoeae	WHO K #3	Zoliflodacin-selected colony #3 (GyrB D429N)	This study
N. gonorrhoeae	WHO K #7	Zoliflodacin-selected colony #7 (GyrB D429N)	This study
N. gonorrhoeae	WHO K #10	Zoliflodacin-selected colony #10 (GyrB D429N)	This study
N. gonorrhoeae	WHO K #13	Zoliflodacin-selected colony #13 (GyrB D429N)	This study
N. gonorrhoeae	WHO L passaging day 8 mutant		This study

Species	Strain ID	Strain information	Source/reference
N. gonorrhoeae	CDC-0197 passaging day 8 mutant		This study
Staphylococcus aureus	ATCC 29213	Methicillin-sensitive	ATCC
Escherichia coli	ATCC 25922	CLSI QC strain	ATCC
E. coli	BAS901C	MC4100 Δ*lamB106 zab*::Tn5 *lptD4123*, drug-susceptible	Eric Brown[18, 19]
Klebsiella pneumoniae	UMM3	KPC-2	Jean-Denis Docquier[20]
Pseudomonas aeruginosa	ATCC 27853	CLSI QC strain	ATCC
P. aeruginosa	ATCC 35151	*lptE*, Δ*oprM*, Δ*ampC*, drug-susceptible[21]	ATCC
Acinetobacter baumannii	ATCC 19606	Type strain	ATCC

ATCC, American Type Culture Collection; CDC, Centers for Disease Control and Prevention; NCTC, National Collection of Type Cultures; CLSI, Clinical and Laboratory Standard Institute.

Table S15. WGS samples with reference genomes

Strain/genotype	Reference genome ID	GenBank-Submitted WGS ID
**FA19**	GCF_001047225.1	NG2
**ATCC 49226**	GCF_001997645.1	NG16
**FA1090**	GCF_000006845.1	NG17
**WHO G**	GCF_900087785.2	NG18
**WHO K**	GCF_900087865.2	NG19
**WHO L**	GCF_900087875.2	NG20
**WHO M**	GCF_900087615.2	NG21
**H041**	GCF_900087815.2	NG22
**CDC-0914, WHO Z**	GCF_900087715.2	NG23
**WHO Q, G7944**	GCF_900411645.1	NG24
**CDC-0197, 12AZI_T_003**	GCA_042026415.1	NG25
**WHO F**	GCF_900087635.2	NG26
**MS11**	GCA_000156855.2	NG27
**F89**	GCF_900087685.2	NG28
**FA19 *porB*$_{\mathrm{FA1090}}$(wt)**	GCF_001047225.1	NG8
**FA19 *porB*$_{\mathrm{FA1090}}$(G120K)**	GCF_001047225.1	NG13
**FA19 *penA4 mtrR171 porB*$_{\mathrm{FA1090}}$(wt)**	GCF_001047225.1	NG12
**FA19 *penA4 mtrR171 porB*$_{\mathrm{FA1090}}$(G120K)**	GCF_001047225.1	NG15
**FA19 *penA4 mtrR171 porB*$_{\mathrm{FA1090}}$(ΔG120ΔA121)**	GCF_001047225.1	NG5
**FA19 *mtrR171* (WV30)**	GCF_001047225.1	NG11
**FA6140 (*mtrR171 penA4 penB ponA1*)**	GCF_001047255.1	NG7
**WV22 (FA6140 *mtrD*::kan)**	GCF_001047255.1	NG9
**WHO K**	GCF_900087865.2	WGS#5_NG1
**WHO K zoliflodacin FoR Colony 3**	GCF_900087865.2	WGS#5_NG2
**WHO K zoliflodacin FoR Colony 7**	GCF_900087865.2	WGS#5_NG3
**WHO K zoliflodacin FoR Colony 10**	GCF_900087865.2	WGS#5_NG4
**WHO K zoliflodacin FoR Colony 13**	GCF_900087865.2	WGS#5_NG5
**WHO L**	GCF_900087875.2<br>GCF_040374935.1	WGS2025_NG-1
**WHO L passage, day 8 mutant**	GCF_900087875.2	WGS2025_NG-2m
**CDC-0197**	GCA_042026415.1	WGS2025_NG-3
**CDC-0197 passage, day 8 mutant**	GCA_042026415.1	WGS2025_NG-4m

Table S16. Crystallographic data collection and model refinement statistics.

	**tPBP2$^{35/02}$-12**	**tPBP2$^{35/02}$-15**	**tPBP2^{H041}-21**
**Data collection**			
Resolution range	38.7–2.6 (2.64-2.60)	38.9-1.89 (1.93-1.90)	40.79-2.10 (2.18-2.10)
Space group	P $2_12_12_1$	P $2_12_12_1$	P $2_12_12_1$
Unit cell *a, b, c* (Å)	50.5, 60.2, 110.3	50.6, 60.7, 110.3	49.9, 60.1, 111.0
Total reflections	76,046	197,367	118,798
Unique reflections	10,605 (518)	27,713 (1,362)	20,048 (5,264)
Multiplicity	7.2 (7.3)	7.1 (7.4)	5.9 (5.6)
Completeness (%)	99.4 (100.0)	99.7 (99.9)	99.4 (99.7)
Mean I/sigma(I)	15.9 (3.9	21.8 (2.7)	7.6 (4.9)
R-merge	0.198 (0.734)	0.109 (0.771)	0.181 (0.401)
R-pim	0.080 (0.291)	0.044 (0.303)	0.082 (0.187)
$CC_{1/2}$	0.971 (0.805)	0.992 (0.802)	0.929 (0.885)
**Refinement**			
R-factor (%)	17.8	17.4	19.3
R-work (%)	17.5 (22.7)	17.3 (21.3)	19.1
R-free (%)	23.2 (29.4)	20.5 (26.3)	23.6
No. of non-hydrogen protein atoms	2,506	2,471	2,442
No. of ligand atoms	39	40	43
No. of waters	19	122	77
RMSDs from ideal stereochemistry:			
RMS (bonds)	0.008	0.009	0.109
RMS (angles)	1.57	1.58	1.74
Ramachandran plot:			
Favored (%)	96.9	98.1	98.4
Allowed (%)	2.5	1.2	1.6
Outliers (%)	0.6	0.6	0.0
B-factors:			
Mean B-factor (all atoms) (Å^2)	29.9	26.1	21.3
Macromolecules (Å^2)	29.7	25.8	21.2
Ligands (Å^2)	42.0	30.8	26.4
Solvent (Å^2)	19.0	29.5	21.5
PDB code	9MD0	9MCZ	9Z5T

Values in parentheses correspond to the outer shell of data for each dataset

Table S17. Liquid chromatography parameters in the final bioanalytical method for boro-PBPi **21**.

**Parameter**	**Value**	
**Ion Mode**	Positive	
**Mobile Phase A**	H_2O + 0.5% formic acid	
**Mobile Phase B**	ACN + 0.5% formic acid	
**Weak needle wash**	90/10 H_2O/MeOH + 0.02%NH_4OH (pH 10)	
**Strong needle wash**	10/90 H_2O/ACN	
**Seal wash**	90/10 H_2O/ACN	
**Column**	Waters Acquity Premier BEH C18 50*2.1mm, 1.7 μm	
**Column Temperature (°C)**	25	
**Auto-sampler Temperature (°C)**	5	
**Injection volume (μL)**	7.5	
**Gradient**		
**Time (min)**	Flow (mL/min)	%MPB
**0**	0.5	15
**0.2**	0.5	15
**0.9**	0.5	30
**1.6**	0.5	95
**2**	0.5	95
**2.01**	0.5	15
**2.5**	0.5	15

Table S18. MS/MS settings in the final bioanalytical method for boro-PBPi **21**.

**Source temperature (°C)**	150
**Desolvation Temperature (°C)**	500
**Cone Gas flow (L/h)**	60
**Desolvation gas flow (L/min)**	1000

Compound	Q1(m/z)	Q3(m/z)	Collision Energy(V)	Cone Voltage (V)
**Boro-PBPi 21**	647.04	629.1	16	20
**Levofloxacin (IS)**	362.2	261.2	20	26

IS = internal standard

Table S19. Validation summary for the boro-PBPi **21** bioanalytical method in rat plasma.

Parameter	Acceptance criteria	Pass/Fail
**Linearity**	R^2 value ≥ 0.98	Passed
**Sensitivity**	± 20% bias (with less than 20% CV)	Passed
**Selectivity**	LLOQ level: ± 20% bias<br>Blank: analyte signal ≤ 20% of the average analyte signal in LLOQ samples<br>DB: analyte signal ≤ 20% of the average analyte signal in LLOQ samples; IS signal ≤ 5% the average IS signal in LLOQ samples.	Passed
**Accuracy and precision**	± 15% bias (with less than 15% CV)	Passed
**Dilution factor**	± 15% bias (with less than 15% CV)	Passed for 10X
**Benchtop stability**	± 15% bias (with less than 15% CV)	Passed for 3.5 hours at RT
**Freeze/Thaw stability**	± 15% bias (with less than 15% CV)	Passed for 4 cycles between -80 °C and RT
**Reinjection stability**	± 15% bias (with less than 15% CV)	Passed for 49 hours at autosampler temperature
**Hemolysis**	± 15% bias (with 15% CV)	Passed for 2% hemolyzed plasma
**Interference to IS from analyte**	≤ 5% of the average IS signal	0%
**Matrix effect**		1.11 (enhancement)
**Recovery**		45.6%

Blank: samples with no analyte, but IS was added.

Double blank: samples with no analyte and IS.

Raw images of gels shown in Figure S3.

ATCC 49226 membrane

CRO (μM)

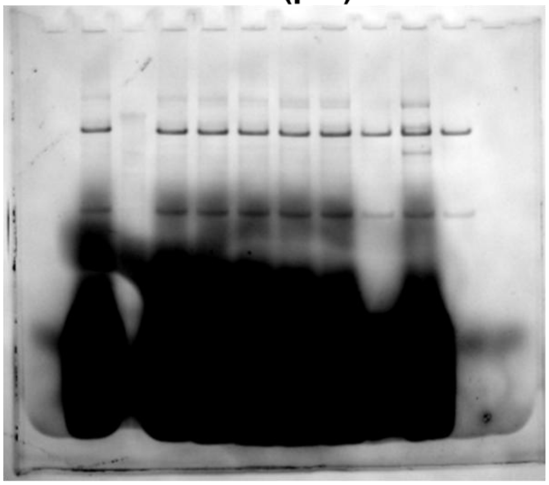

Boro-PBPi 21 (μM)

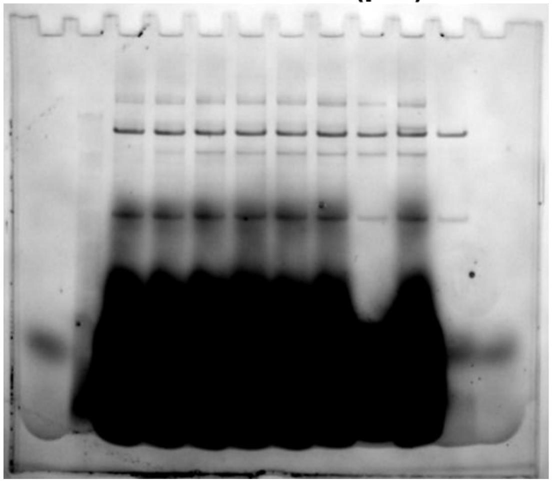

H041 membrane

CRO (μM)

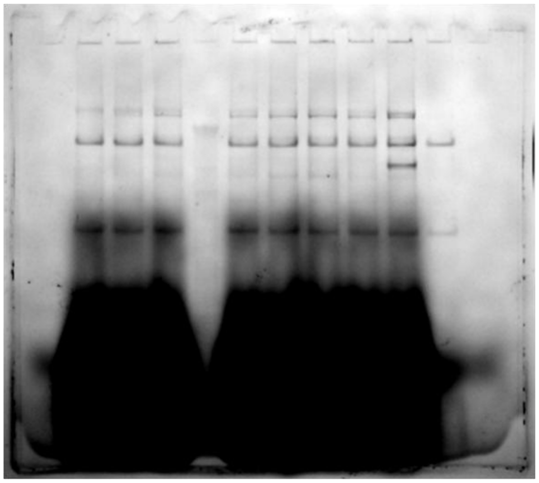

Boro-PBPi 21 (μM)

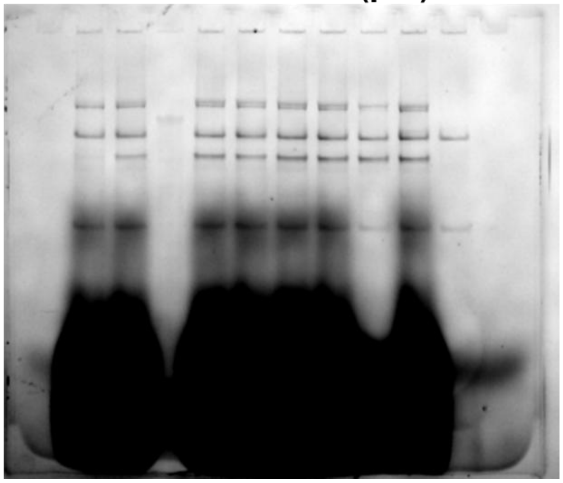

REFERENCES

1. Lopez-Arguello S*, et al.* Penicillin-binding protein occupancy dataset for 18 beta-lactams and 4 beta-lactamase inhibitors in *Neisseria gonorrhoeae*. *Microbiol Spectr* 11, e0069223 (2023). DOI: 10.1128/spectrum.00692-23.

2. Stefanova ME, Tomberg J, Olesky M, Holtje JV, Gutheil WG, Nicholas RA. *Neisseria gonorrhoeae* penicillin-binding protein 3 exhibits exceptionally high carboxypeptidase and beta-lactam binding activities. *Biochemistry* 42, 14614-14625 (2003). DOI: 10.1021/bi0350607.

3. Demczuk W*, et al. Neisseria gonorrhoeae* sequence typing for antimicrobial resistance, a novel antimicrobial resistance multilocus typing scheme for tracking global dissemination of *N. gonorrhoeae* strains. *J Clin Microbiol* 55, 1454-1468 (2017). DOI: 10.1128/JCM.00100-17.

4. Liu H, Vidyaprakash E, Schmerer MW, Pham DC, St Cyr S, Kersh EN. A culture collection of 50 *Neisseria gonorrhoeae* isolates. *Microbiol Resour Announc* 9, (2020). DOI: 10.1128/MRA.00699-20.

5. Amin K, Dannenfelser RM. In vitro hemolysis: guidance for the pharmaceutical scientist. *J Pharm Sci* 95, 1173-1176 (2006). DOI: 10.1002/jps.20627.

6. Tilmant K*, et al.* The automated micronucleus assay for early assessment of genotoxicity in drug discovery. *Mutat Res* 751, 1-11 (2013). DOI: 10.1016/j.mrgentox.2012.10.011.

7. Nishimuta H, Houston JB, Galetin A. Hepatic, intestinal, renal, and plasma hydrolysis of prodrugs in human, cynomolgus monkey, dog, and rat: implications for in vitro-in vivo extrapolation of clearance of prodrugs. *Drug Metab Dispos* 42, 1522-1531 (2014). DOI: 10.1124/dmd.114.057372.

8. Maness MJ, Sparling PF. Multiple antibiotic resistance due to a single mutation in *Neisseria gonorrhoeae*. *J Infect Dis* 128, 321-330 (1973). DOI: 10.1093/infdis/128.3.321.

9. Black WJ, Schwalbe RS, Nachamkin I, Cannon JG. Characterization of *Neisseria gonorrhoeae* protein II phase variation by use of monoclonal antibodies. *Infect Immun* 45, 453-457 (1984). DOI: 10.1128/iai.45.2.453-457.1984.

10. Unemo M*, et al.* The novel 2016 WHO *Neisseria gonorrhoeae* reference strains for global quality assurance of laboratory investigations: phenotypic, genetic and reference genome characterization. *The Journal of antimicrobial chemotherapy* 71, 3096-3108 (2016). DOI: 10.1093/jac/dkw288.

11. Bhat KS*, et al.* The opacity proteins of *Neisseria gonorrhoeae* strain MS11 are encoded by a family of 11 complete genes. *Mol Microbiol* 5, 1889-1901 (1991). DOI: 10.1111/j.1365-2958.1991.tb00813.x.

12. Eyre DW*, et al.* Gonorrhoea treatment failure caused by a *Neisseria gonorrhoeae* strain with combined ceftriaxone and high-level azithromycin resistance, England, February 2018. *Euro Surveill* 23, (2018). DOI: 10.2807/1560-7917.ES.2018.23.27.1800323.

13. Unemo M, Golparian D, Nicholas R, Ohnishi M, Gallay A, Sednaoui P. High-level cefixime- and ceftriaxone-resistant *Neisseria gonorrhoeae* in France: novel *penA* mosaic allele in a successful international clone causes treatment failure. *Antimicrob Agents Chemother* 56, 1273-1280 (2012). DOI: 10.1128/AAC.05760-11.

14. Connolly KL, Eakin AE, Gomez C, Osborn BL, Unemo M, Jerse AE. Pharmacokinetic data are predictive of in vivo efficacy for cefixime and ceftriaxone against susceptible and

resistant *Neisseria gonorrhoeae* strains in the gonorrhea mouse model. *Antimicrob Agents Chemother* 63, (2019). DOI: 10.1128/AAC.01644-18.

15. Olesky M, Zhao S, Rosenberg RL, Nicholas RA. Porin-mediated antibiotic resistance in *Neisseria gonorrhoeae*: ion, solute, and antibiotic permeation through PIB proteins with *penB* mutations. *J Bacteriol* 188, 2300-2308 (2006). DOI: 10.1128/JB.188.7.2300-2308.2006.

16. Veal WL, Nicholas RA, Shafer WM. Overexpression of the MtrC-MtrD-MtrE efflux pump due to an *mtrR* mutation is required for chromosomally mediated penicillin resistance in *Neisseria gonorrhoeae*. *J Bacteriol* 184, 5619-5624 (2002). DOI: 10.1128/JB.184.20.5619-5624.2002.

17. Faruki H, Sparling PF. Genetics of resistance in a non-beta-lactamase-producing gonococcus with relatively high-level penicillin resistance. *Antimicrob Agents Chemother* 30, 856-860 (1986). DOI: 10.1128/AAC.30.6.856.

18. Vulic M, Kolter R. Alcohol-induced delay of viability loss in stationary-phase cultures of *Escherichia coli*. *J Bacteriol* 184, 2898-2905 (2002). DOI: 10.1128/JB.184.11.2898-2905.2002.

19. Sampson BA, Misra R, Benson SA. Identification and characterization of a new gene of *Escherichia coli* K-12 involved in outer membrane permeability. *Genetics* 122, 491-501 (1989). DOI: 10.1093/genetics/122.3.491.

20. Smith Moland E*, et al.* Plasmid-mediated, carbapenem-hydrolysing beta-lactamase, KPC-2, in *Klebsiella pneumoniae* isolates. *The Journal of antimicrobial chemotherapy* 51, 711-714 (2003). DOI: 10.1093/jac/dkg124.

21. Shen X*, et al.* Defects in efflux (*oprM*), beta-lactamase (*ampC*), and lipopolysaccharide transport (*lptE*) genes mediate antibiotic hypersusceptibility of *Pseudomonas aeruginosa* strain Z61. *Antimicrob Agents Chemother* 63, (2019). DOI: 10.1128/AAC.00784-19.
