# Supplementary material for: A penicillin-binding protein inhibitor series to target drug-resistant Neisseria gonorrhoeae
Source: Nat Microbiol. 2026 Apr 8;11(5):1348–60. doi: 10.1038/s41564-026-02309-3 (PMC13171604; doi:10.1038/s41564-026-02309-3)
Supplement: Supplementary file 2 — Reporting Summary [file 41564_2026_2309_MOESM2_ESM.pdf]

nature portfolio

Corresponding author(s): Tsuyoshi Uehara, Stephen Condon

Last updated by author(s): Jan 26, 2026

Reporting Summary

Nature Portfolio wishes to improve the reproducibility of the work that we publish. This form provides structure for consistency and transparency in reporting. For further information on Nature Portfolio policies, see our Editorial Policies and the Editorial Policy Checklist.

Statistics

For all statistical analyses, confirm that the following items are present in the figure legend, table legend, main text, or Methods section.

n/a	Confirmed	
☐	☒	The exact sample size (n) for each experimental group/condition, given as a discrete number and unit of measurement
☐	☒	A statement on whether measurements were taken from distinct samples or whether the same sample was measured repeatedly
☐	☒	The statistical test(s) used AND whether they are one- or two-sided<br>*Only common tests should be described solely by name; describe more complex techniques in the Methods section.*
☐	☒	A description of all covariates tested
☐	☒	A description of any assumptions or corrections, such as tests of normality and adjustment for multiple comparisons
☐	☒	A full description of the statistical parameters including central tendency (e.g. means) or other basic estimates (e.g. regression coefficient) AND variation (e.g. standard deviation) or associated estimates of uncertainty (e.g. confidence intervals)
☐	☒	For null hypothesis testing, the test statistic (e.g. F, t, r) with confidence intervals, effect sizes, degrees of freedom and P value noted<br>*Give P values as exact values whenever suitable.*
☒	☐	For Bayesian analysis, information on the choice of priors and Markov chain Monte Carlo settings
☒	☐	For hierarchical and complex designs, identification of the appropriate level for tests and full reporting of outcomes
☒	☐	Estimates of effect sizes (e.g. Cohen's d, Pearson's r), indicating how they were calculated

Our web collection on statistics for biologists contains articles on many of the points above.

Software and code

Policy information about availability of computer code

Data collection	X-ray diffraction data were collected at the SER-CAT 22-ID beamline at the Advanced Photon Source in Argonne, IL, USA.
Data analysis	HKL2000; X-ray diffraction data processing<br>PHENIX 1.18.2-3874 Industrial Consortium Map sharpening; model refinement and validation<br>COOT 0.9.8.95 MRC Laboratory of Molecular Biology, Computational Structural Biology Group; Model building<br>REFMAC5 5.7.0009 MRC Laboratory of Molecular Biology, Computational Structural Biology Group; Refinement of Macromolecular Structures<br>ChemDraw Professional 22 and 23; chemical analysis<br>Molecular Operating Environment (MOE) 2022 and 2024; Structure/ligand-based inhibitor design, SAR, modeling, computational chemistry<br>Microsoft 365 (Excel, PowerPoint); Data analyses, tables, figures<br>GraphPad Prism 9 and 10; Data plotting and analyses, nonlinear regression, Anova analysis<br>Phoenix WinNonlin 8.3; Pharmacokinetics analysis<br>Geneious Prime 2022, 2023, 2024, 2025, and 2026; DNA and protein sequence analysis including genome sequence analysis<br>Waters Analyst 1.7.1 and SCIEX OS 2.0.0.45330; UPLC-MS/MS data acquisition

For manuscripts utilizing custom algorithms or software that are central to the research but not yet described in published literature, software must be made available to editors and reviewers. We strongly encourage code deposition in a community repository (e.g. GitHub). See the Nature Portfolio guidelines for submitting code & software for further information.

Data

Policy information about availability of data

All manuscripts must include a data availability statement. This statement should provide the following information, where applicable:

- Accession codes, unique identifiers, or web links for publicly available datasets
- A description of any restrictions on data availability
- For clinical datasets or third party data, please ensure that the statement adheres to our policy

The data that support the findings of this study are included in this published article and its supplementary information. Structural data have been deposited in the PDB under the accession codes 9MD0 (tPBP235/02-12), 9MCZ (tPBP235/02-15) and 9Z5T (tPBP2H041-21). Raw sequence reads obtained from N. gonorrhoeae strains in this study were deposited in GenBank under BioProject accession number PRJNA1353147.

Research involving human participants, their data, or biological material

Policy information about studies with human participants or human data. See also policy information about sex, gender (identity/presentation), and sexual orientation and race, ethnicity and racism.

Reporting on sex and gender	N/A, neither human participants nor human data are reported in this manuscript.
Reporting on race, ethnicity, or other socially relevant groupings	N/A
Population characteristics	N/A
Recruitment	N/A
Ethics oversight	N/A

Note that full information on the approval of the study protocol must also be provided in the manuscript.

Field-specific reporting

Please select the one below that is the best fit for your research. If you are not sure, read the appropriate sections before making your selection.

☒ Life sciences ☐ Behavioural & social sciences ☐ Ecological, evolutionary & environmental sciences

For a reference copy of the document with all sections, see nature.com/documents/nr-reporting-summary-flat.pdf

Life sciences study design

All studies must disclose on these points even when the disclosure is negative.

Sample size	Murine PK studies (n=3 mice/group); Murine in vivo efficacy studies for boro-PBPi 18 (n=5 mice/group) and 21 (n=10 mice/group); Rat PK study (n=3 rats/group)
Data exclusions	No data were excluded from analyses.
Replication	The experiments were not replicated, because the sufficient samples of each group for examining significant difference were included in each study.
Randomization	Mice were randomized for drug/vehicle treatment.
Blinding	The investigators were not blinded as mice and samples were tracked from treatments and sampling to bioanalysis (colony counting or LC-MS/MS).

Reporting for specific materials, systems and methods

We require information from authors about some types of materials, experimental systems and methods used in many studies. Here, indicate whether each material, system or method listed is relevant to your study. If you are not sure if a list item applies to your research, read the appropriate section before selecting a response.

Materials & experimental systems

n/a	Involved in the study	
☒	☐	Antibodies
☐	☒	Eukaryotic cell lines
☒	☐	Palaeontology and archaeology
☐	☒	Animals and other organisms
☒	☐	Clinical data
☒	☐	Dual use research of concern
☒	☐	Plants

Methods

n/a	Involved in the study	
☒	☐	ChIP-seq
☒	☐	Flow cytometry
☒	☐	MRI-based neuroimaging

Eukaryotic cell lines

Policy information about cell lines and Sex and Gender in Research

Cell line source(s)	ATCC: MRC-5 (ATCC CCL-171), HeLa (ATCC CCL-2), 3T3 (ATCC CCL-92), SKOV-3 (ATCC HTB-77), CHO-K1 (ATCC CCL-61)
Authentication	None of the cell lines was authenticated.
Mycoplasma contamination	None of the cell lines was checked.
Commonly misidentified lines (See ICLAC register)	MRC-5, HeLa, and 3T3 are listed. The source of these cell lines used was ATCC as described above.

Animals and other research organisms

Policy information about studies involving animals; ARRIVE guidelines recommended for reporting animal research, and Sex and Gender in Research

Laboratory animals	Female Balb/c mice for PK studies, 7–9 weeks old, received from Vital River, Zhejiang, China<br>Male CD-1 mice for PK studies, 7–9 weeks old, received from Vital River, Zhejiang, China<br>Female Balb/c mice for efficacy studies, 5–6 weeks old, received from BioLASCO Taiwan<br>Female NCI BALB/c mice for efficacy studies, 6–7 weeks old, received from Charles River Laboratories<br>Male Sprague Dawley rats for PK studies, 5–7 weeks old, received from Hilltop Labs Animals, Inc.
Wild animals	N/A
Reporting on sex	Female mice was used for in vivo efficacy studies because only vaginal infection models have been established to to test compound acitvity against N. gonorrhoeae.
Field-collected samples	N/A
Ethics oversight	Animal experiments were conducted at the Uniformed Services University (USU) of the Health Sciences (Bethesda, MD, USA), the BioDuro-Sundia DMPK group (Jiangsu, China), Eurofins Pharmacology Discovery Services (New Taipei City, Taiwan), and QPS, LLC (Newark, DE, USA). Each facility is accredited by the Association for the Assessment and Accreditation of Laboratory Animal Care (AAALAC) and is provided assurance from the National Institutes of Health Public Health Services' Office of Laboratory Animal Welfare (OLAW), with protocols approved by the Institutional Animal Care and Use Committees (IACUC). The IACUC-approved protocol numbers were BioDuro BD-202102114 (PK studies for boro-PBPi 18), BioDuro BDW-2201-0007 (PK studies for boro-PBPi 21), PDS IM005-07302018 (efficacy studies for boro-PBPi 18), USU MIC23-759 (efficacy studies for boro-PBPi 21), and QPS Number 005 (rat PK studies).

Note that full information on the approval of the study protocol must also be provided in the manuscript.

Plants

Seed stocks	N/A
Novel plant genotypes	N/A
Authentication	N/A
